# 5,7-Dimethoxychroman-3-yl 4‑methoxybenzoate Has a Unique Effect upon the Modulation of Mutant Cardiac Muscle Thin Filament Function and Dynamics due to Phosphorylation of Troponin I

**DOI:** 10.1021/acsptsci.5c00156

**Published:** 2025-04-15

**Authors:** Zeyu Yang, Alice M. Sheehan, Mary Papadaki, Andrew E. Messer, Brian S. J. Blagg, Ian R. Gould, Steven B. Marston

**Affiliations:** † Institute of Chemical Biology, Molecular Sciences Research Hub and Department of Chemistry, Molecular Sciences Research Hub, 4615 Imperial College London, London W12 0BZ, United Kingdom; ‡ NHLI, 4615 Imperial College London, London W12 ONN, United Kingdom; § 6111 The University of Notre Dame, 305 McCourtney, Notre Dame, Indiana 46556, United States; ∥ Department of Chemistry, Molecular Sciences Research Hub and Institute of Chemical Biology, Molecular Sciences Research Hub, 4615 Imperial College London, London W12 0BZ, United Kingdom; ⊥ NHLI, 4615 Imperial College London, London W12 0NN, United Kingdom

**Keywords:** cardiac troponin I, cardiac troponin C, phosphorylation, molecular dynamics, thin filament proteins

## Abstract

Mutations in thin filament proteins that cause cardiomyopathy commonly cause an uncoupling of the relationship between the phosphorylation of troponin I and reduced Ca^2+^ sensitivity. Previously we showed that small molecules related to EGCG were able to restore the native response to mutant thin filaments *in vitro* and in MD simulations. However, 5,7-dimethoxychroman-3-yl 4-methoxybenzoate (compound **7**) has an opposite effect—it causes mutant thin filament Ca^2+^ sensitivity to increase when cTroponin I is phosphorylated. In MD simulations of troponin with the TNNC1 G159D DCM mutation, we observed that compound **7** has unique effects upon troponin dynamics. Global parameters, such as interdomain hinge angle and Troponin C helix A/B angle distributions tend to be independent of phosphorylation unlike the phosphorylation-dependent changes observed with G159D alone or G159D plus recouplers such as silybin B. CCPTraj and Cluster Analysis suggest a novel preferred binding region between the extreme N terminus of cTroponin C and the switch peptide of cTroponin I.

## Graphical abstract

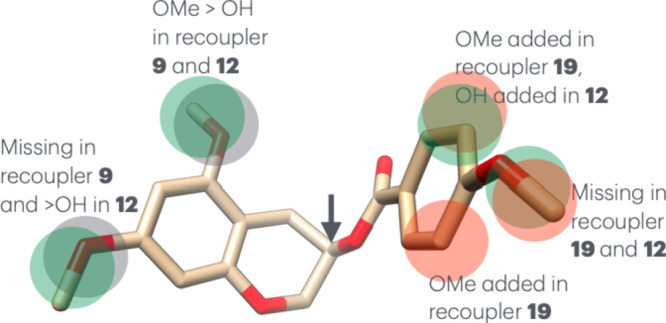

Phosphorylation of cardiac troponin by PKA plays a key role in the heart’s response to adrenergic stimulation. Phosphorylation at Serines 22 and 23 of cTroponin I leads to a 2-fold decrease in Ca^2+^-sensitivity of the myofilaments linked to a 2-fold increase in the rate of Ca^2+^-release from troponin and hence quicker relaxation.^1–4^ This process is called lusitropy and together with inotropy and chronotropy makes up the normal response to adrenergic activation.

It is now well established that mutations in the thin filament proteins (actin, tropomyosin, troponin I, C or T) that cause cardiomyopathy—both HCM and DCM—lack this response; PKA phosphorylation does not change Ca^2+^-sensitivity and lusitropy is suppressed.^5,6^ Suppression of lusitropy contributes to the cardiomyopathic disease phenotype and in some cases of dilated cardiomyopathy, notably ACTC E361G, can be causative of heart failure.^7,8^

We have researched small molecules structurally related to silybin and EGCG that can restore lusitropy and phosphorylation-dependent modulation of Ca^2+^-sensitivity. In our initial study of 40 compounds, we found 23 that could restore the phosphorylation-dependent Ca^2+^-sensitivity shift, called “recoupling”, and therefore had therapeutic potential.^9^ Five of these compounds were examined by molecular dynamics simulations to elucidate the mechanism of recoupling^10^ and three of them—EGCG, silybin B, and resveratrol—were also shown to restore lusitropy in cardiomyocytes.

The rest of the compounds had no effect except for one compound that, remarkably, had the reverse effect of recoupling: 5,7-dimethoxychroman-3-yl 4-methoxybenzoate (compound **7**) at 100 μM had no effect on the modulation of Ca^2+^ sensitivity by phosphorylation in wild-type thin filaments. However, in thin filaments containing a cardiomyopathy mutation, it caused the phosphorylated species to have a higher Ca^2+^-sensitivity than the unphosphorylated thin filaments.^9^ This reverse-recoupling property is all the more remarkable since several closely related compounds act as normal recouplers.

In this study, we have investigated the functional effects of compound **7** upon thin filaments and we have extended our previous molecular dynamics simulations of the effect of troponin I phosphorylation and ligand binding on cardiac troponin structure and dynamics in order to elucidate the molecular mechanism of the reverse effect of compound **7** in comparison with compounds that are normal recouplers.

## Results and Discussion

### 5,7-Dimethoxychroman-3-yl 4-methoxybenzoate (Compound **7**)

Compound **7** is one of a series of 26 analogues of epigallocatechin 3 gallate (EGCG) synthesized by Khandelwahl and Blagg^11^ that were tested blind for their ability to reverse uncoupling in mutant thin filaments.^9^ It is a chiral compound and has two possible orientations (s and r) although the species tested was presumably a racemic mixture.

Compound **7** has close similarities with two compounds that are recouplers (**9** and **19**) and one compound, **12**, that was inactive; Figure 1 shows how they differ from compound **7** (see also Figure S3). The differences in compound **9** are in the chromane ring while the differences for compound **19** are in the benzoate ring, while compound **12** shows differences in both. Compound **7** has three OMe substitutions whose positions seem important for function

**Figure 1:**
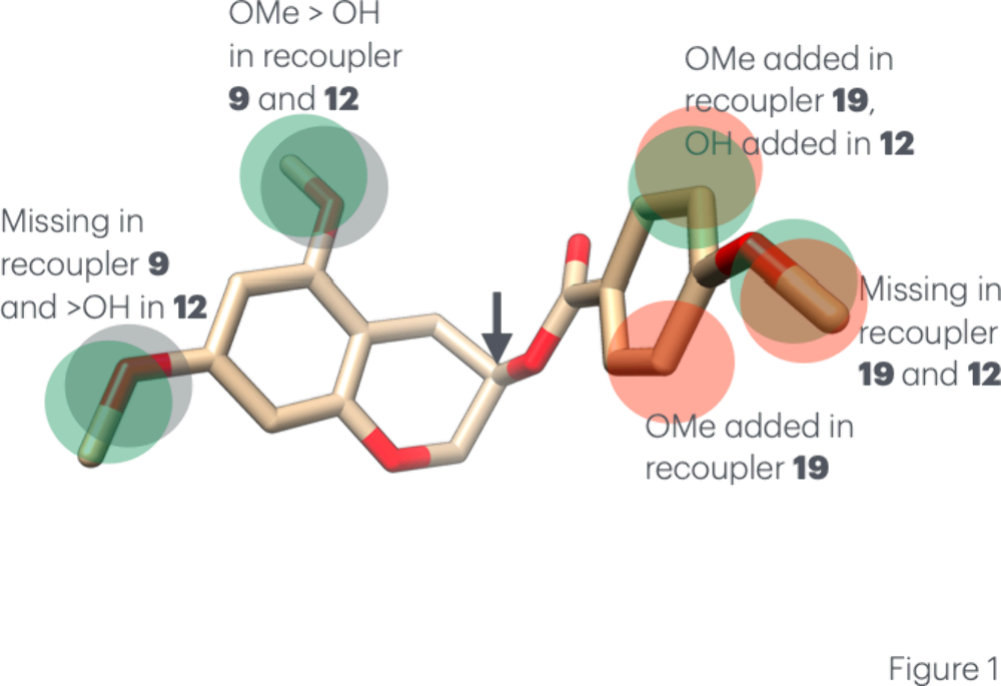
The calculated preferred solution conformation of s5,7-dimethoxychroman-3-yl 4-methoxybenzoate (compound **7**). Arrow indicates the chiral center. Also shown are the differences in compounds **9** (gray), **12** (green), and **19** (pink) (see Table 2 and Figure S3).

### Functional Properties of Compound **7**

Compound **7** was tested for its effect upon thin filament Ca^2+^ regulation with phosphorylated and unphosphorylated troponin I using an *in vitro* motility assay.

In the presence of compound **7**, Ca^2+^ activation of wild-type thin filaments is normal and the Ca^2+^ sensitivity shift on phosphorylation is not changed (Figure 2A and Table 1). This contrasts with the parent molecule, EGCG, which is clearly a desensitizer as previously noted.^12^ This effect can also be demonstrated in a single point assay where the difference in motility between phosphorylated and unphosphorylated at 0.075 μM Ca^2+^ (i.e around EC_50_ for the unphosphorylated state) is observed to be the same independent of compound **7** concentration (Figure 2B).

**Table 1:**
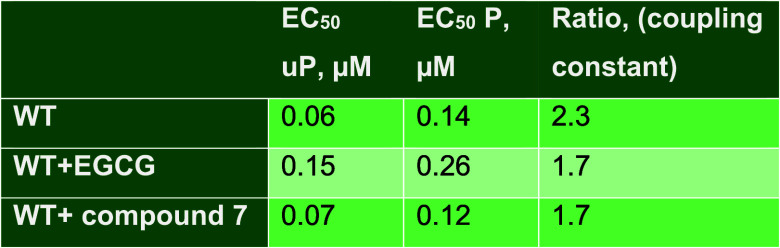
Effects of EGCG and Compound **7** on the Phosphorylation Dependence of Wild-Type Thin Filament Ca^2+^ Sensitivity Measured by an In Vitro Motility Assay^a^

a Values are the means of two separate Ca^2+^-curve measurements.

**Figure 2:**
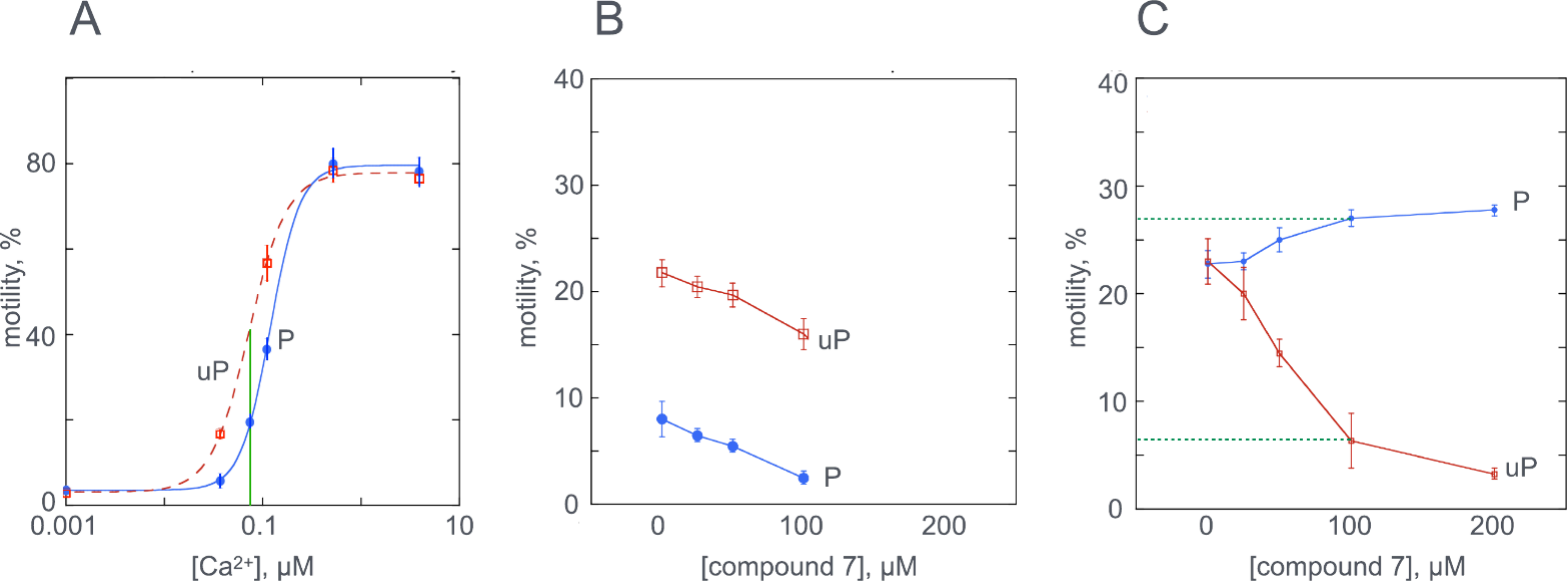
Phosphorylation dependence of Ca^2+^ regulation in the presence of compound **7**. Fraction (%) of thin filaments moving measured by IVMA; filament velocity mirrors % motile and is shown in supplement 1A. (A) Wild-type thin filament Ca^2+^ activation curve in the presence of 100 μM compound **7**. Phosphorylated (blue) and unphosphorylated (red); phosphorylation reduces the Ca^2+^ sensitivity. Calculated EC_50_ values are given in Table 1. Green line is at 0.075 μM Ca^2+^ as used for single point assay. (B) Single point assay dose–response curve for phosphorylated (blue) and unphosphorylated (red) wild-type thin filaments in the presence of zero to 100 μM compound **7**. (C) Single point assay dose–response curve for phosphorylated (blue) and unphosphorylated (red) thin filaments containing E180G tropomyosin. Dotted green lines indicate motility at 100 μM compound **7**.

In contrast, in thin filaments containing the uncoupling TPM1 E180G mutation, the single point assay shows that the difference in % filaments motile between phosphorylated and unphosphorylated thin filaments at 0.075 μM Ca^2+^ is zero, indicating uncoupling due to the mutation. Upon adding compound **7** the motility is increased on phosphorylation and decreased in the unphosphorylated state indicating that in the presence of compound **7** Ca^2+^ sensitivity increases upon phosphorylation. An EC_50_ of 67 μM was calculated. This is the opposite of all other recouplers^9^ as demonstrated by the closely related compounds **9** and **19** as well as the previously studied EGCG and silybin B (see Table 2 and Supplement 1). Previous studies indicate that the recoupling effect of small molecules is independent of the causative mutation and that a full restoration of the effects of troponin I phosphorylation is achieved by all recouplers.^9,12^ We believe the same principles apply to the reverse recoupling effect of compound **7** since this effect is not specific to the TPM1 E180G mutation but was also observed with ACTC E99K (HCM) and TPM E54K (DCM) mutations (see Figure S2)

**Table 2:**
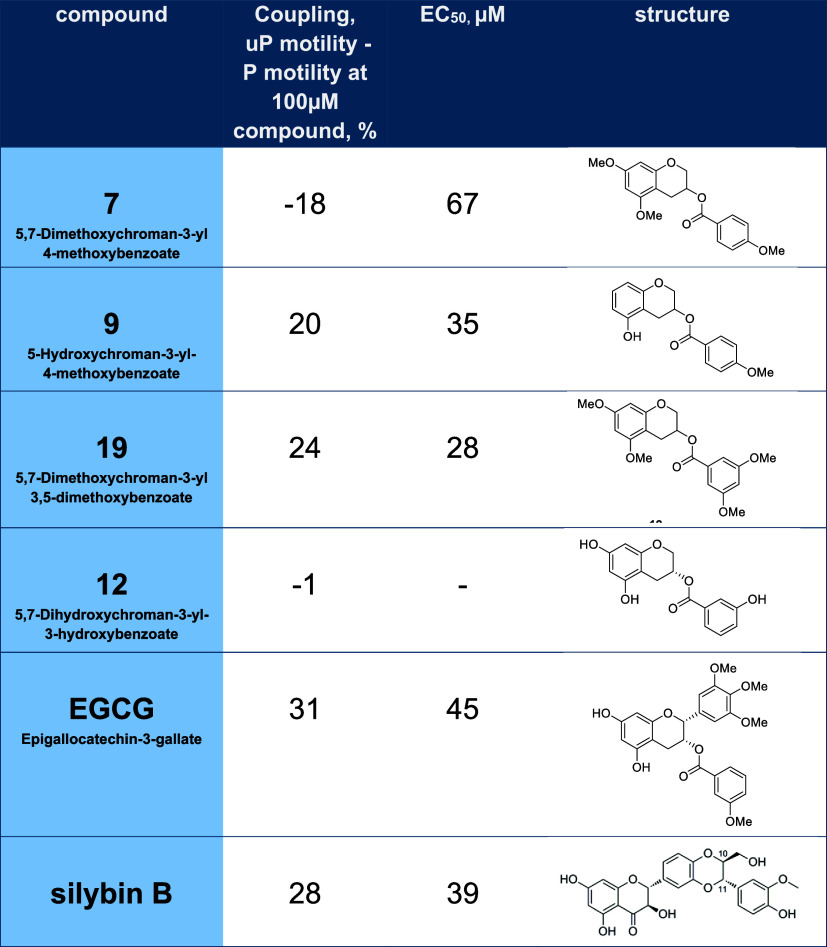
Single Point Assay of Thin Filaments Containing TPM1 E180G Mutation in the Presence of 100 μM Compound **7** Compared with Recouplers and an Inactive Compound (see Figure 2C)^a^

a The calculated EC_50_ and chemical structures are also shown. Differences between % motility for phosphorylated and unphosphorylated forms of thin filaments at 0.075 μM Ca^2+^, 100 μM compound and EC_50_ for the compound are shown. Positive values indicate recoupling (wild-type value is 20–30). Compound **7** is the only compound showing a negative coupling constant (see Figure S1). Includes data from Papadaki et al.^12^ and Sheehan et al.^9^

### Structural and Dynamic Aspects of Compound **7** Interaction with Troponin

To understand how the reverse recoupler compound **7** modulates troponin behavior differently from recouplers at the atomic level, we performed molecular dynamics simulations. Five × 1500 ns simulations were run for each of 8 conditions. We simulated wild-type and TnC G159D DCM mutant troponin in the phosphorylated and unphosphorylated states in the presence of compound **7r** and compound **7s**. It was necessary to simulate both stereoisomers of compound **7** since with other small molecules such as silybin, stereoisomers act differently both functionally and structurally.^13^ We compared the results with our simulations of the “pure” recoupler, silybin B and the parent compound EGCG.

In previous molecular dynamics simulations, we demonstrated that phosphorylation changes the dynamics of wild-type troponin and the uncoupling mutant Troponin C G159D quite differently and that recouplers, such as EGCG, resveratrol and silybin B shift the parameters of mutant thin filaments back to the wild-type values.^10^ We have derived several metrics that are characteristic of the phosphorylated and unphosphorylated state. The distribution of the angle between cTroponin C helices A and B and the angle between the quasi-rigid troponin domains (NcTnC and the ITC domain) may be used to track global effects of small molecules binding.^10^ The distribution of angles is shown in Figure S4 and quantification of the effects are tabulated in Tables 3 and 4. The key results are summarized in Figure 3. Compound **7** evidently affects the phosphorylation-dependent transition in a different way to the recouplers silybin B and EGCG, although it does not act simply as an “anti silybin B” agent, indicating a novel mechanism.

**Table 3:**
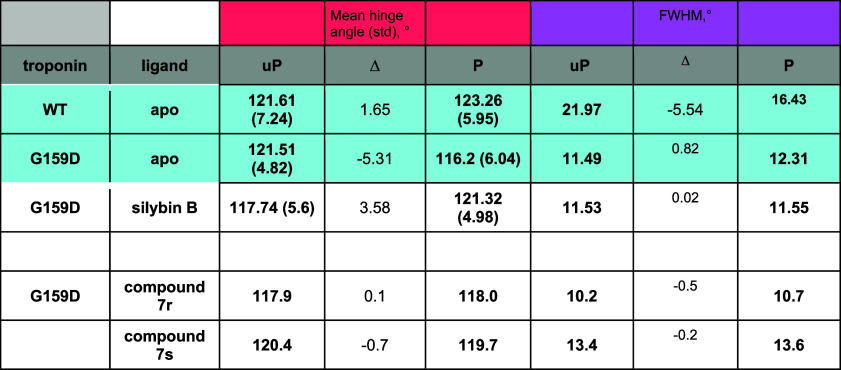
Quantification of the Effects of Phosphorylation and Compound **7** on Troponin Hinge Angle Metrics Compared with Silybin B

a The distributions of hinge angle in the presence of compound **7** and silybin B is shown in Figure S4. Mean hinge angle and fwhm (full width half-maximum) calculated from these data are plotted in the table.

**Table 4:**
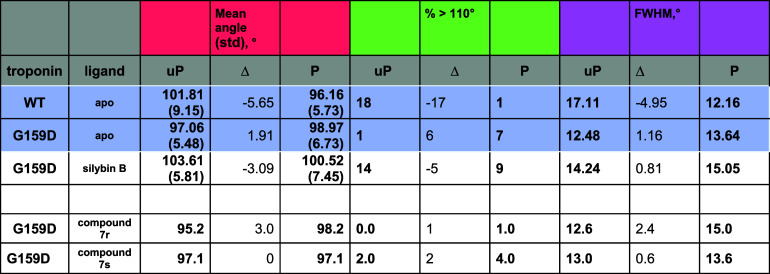
Quantification of the Effects of Phosphorylation and Compound **7** on Troponin A/B Angle Metrics Compared with Silybin B

a The distributions of A/B angle in the presence of compound **7** and silybin B is shown in Figure S4. Mean hinge angle and fwhm (full width half-maximum) calculated from these data are plotted in the table.

**Figure 3:**
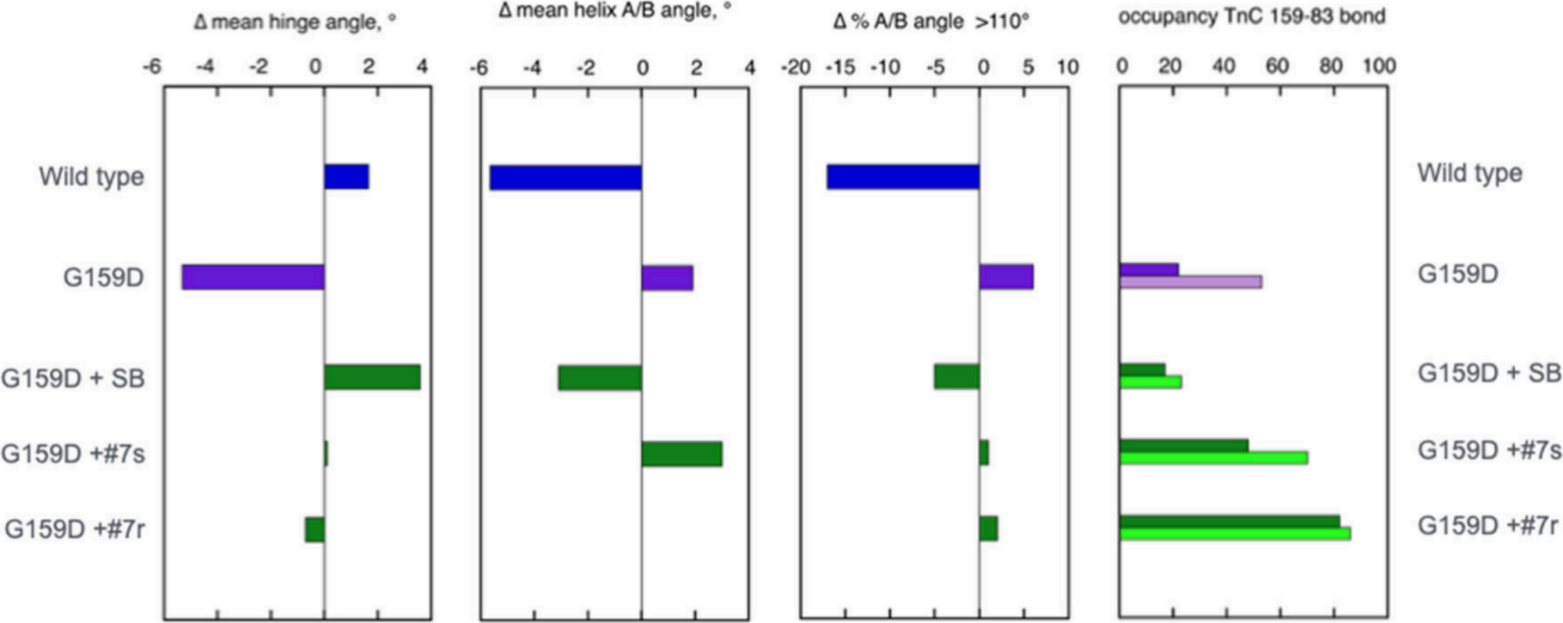
Summary comparison of the effect of phosphorylation of cardiac troponin on molecular dynamics parameters of wild-type and TnC G159D mutant thin filaments and the effects of silybin B and compound **7**. Wild type, blue; G159D, purple; G159D+ ligand, green. Parameters compared are the change in mean hinge angle, change in mean A/B angle and fraction of A/B angles >110 upon phosphorylation and the occupancy of the ionic bond between TnC D159 and K83 from Molecular dynamics simulations. Pale and dark colors indicate phosphorylated and unphosphorylated, respectively. Parameter distribution plots and calculated parameters are shown in Tables 3 and 4 and Figures S4 and S5.

The interdomain hinge angle change upon phosphorylation is positive in wild-type troponin and negative in G159D. The recoupler silybin B restores the positive change to G159D but neither of the stereoisomers of compound **7** shows significant change in the mean angle on phosphorylation. This lack of effect is correlated with the occupancy of the strong bond between Asp159 and Lys83 of TnC, which cross-links the two domains of troponin. Recouplers diminish this bond strongly, but compound **7** has almost no effect on the cross-linking (Figure S5)

The hinge angle differences are reflected in the measurements of the changes in the angle between TnC helices A and B. In wild-type, phosphorylation reduces the angle while the angle is increased in G159D troponin. Silybin B can restore the angle of G159D toward wild-type values, but compound **7** does not; indeed, compound **7s** shows no phosphorylation-dependent change.

Overall, compound **7** seems to suppress phosphorylation dependence of structural dynamics rather than have an effect antagonistic to recouplers. Also, it is notable that there is little difference between the effects of r and s isomers unlike silybin A and B. The effects of compound **7** on troponin dynamics are unique, as are its functional effects. It is not clear how such a global effect could be responsible for the “reverse-recoupling” effect that we have observed, therefore we investigated how compound **7r** and **s** bind to troponin at the atomic level.

### Compound **7** Interaction with Troponin Determined by MD Simulations

Due to the dynamic nature of the troponin molecule, it is not possible to provide a detailed picture of ligand binding, but we can calculate the probabilities of the ligand being in a small number of states and how the balance between states is influenced by phosphorylation, mutation, and ligands.

Initially, we identified the probability of ligand contacts on troponin by using the CPPTraj procedures. Figure 4 plots the probability of interactions between the ligand and each amino acid of the troponin core. The overall result of this calculation is that compound **7r** and compound **7s** have similar contacts with troponin and that silybin B is very different. Silybin B has phosphorylation-dependent contacts with the C terminus of Troponin T (276–278) that compound **7** does not. Compound **7** has very little contact with the N terminus of cTroponin I unlike silybin B. Compound **7r** and **s** both have contacts with the cTroponin I switch peptide region (154–164) that are largely independent of phosphorylation whereas silybin B contacts only occur in unphosphorylated troponin and at a different site (134–141).

**Figure 4:**
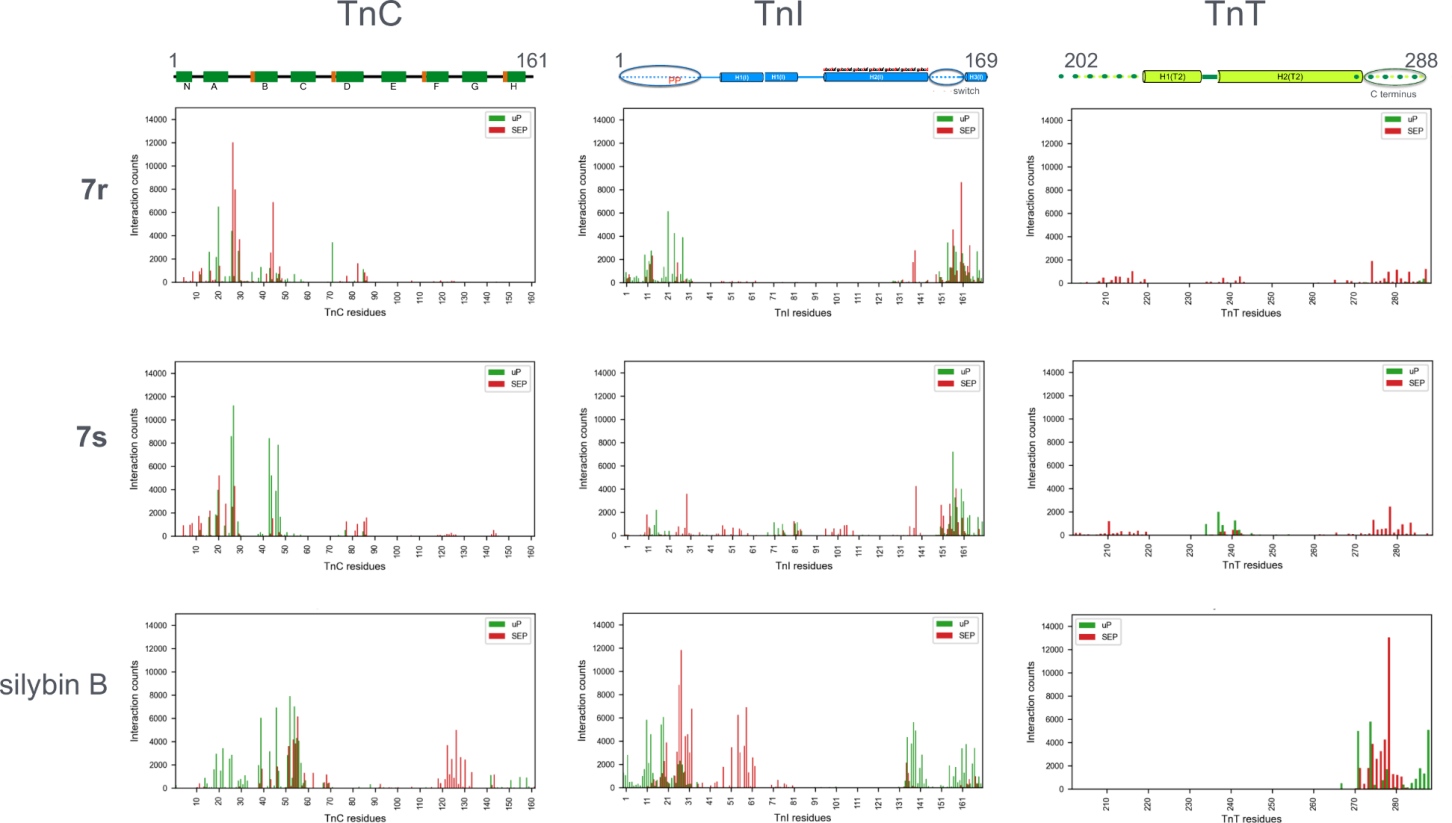
CPPTraj analysis of the probability of ligand contacts with individual amino acids of troponin C (1–161), I (1–169) and T (202–288). 37500 frames were analyzed. Y axis (0–14,000) shows the number of frames where any atom in the ligand is closer than 2.0 Å. Green is unphosphorylated and red is phosphorylated.

The major interactions of compound **7** are with cTroponin C at the extreme N terminus (between 1 and 50, EF hand 1) in contrast to the major interactions of silybin B that center on the 50–60 region (Helix C). Compound **7r** and compound **7s** have different interactions here and different changes on phosphorylation. Thus, compounds **7r** and **s** interact with troponin in a unique way that is consistent with its unique reverse-recoupling property.

CCPTraj calculations are quantitative but one-dimensional and so not very informative of the actual mode of interaction with troponin. This requires 3-dimensional information. One way to identify the most probable methods of binding is cluster analysis. This procedure aims to narrow down the ligand binding regions to a few long-lasting ones that are likely to be the active site. The results of cluster analysis (Figure S6) indicate that one site of interaction predominates and constitutes 90% of compound **7r** and 77% of compound **7s** binding to unphosphorylated G159D compared with 97% for the similar but different site occupied by silybin B. Interestingly the same site is also occupied by compound **7r** and compound **7s** when phosphorylated (78% and 58% respectively) but the silybin B site is much less occupied.

According to the cluster analysis (Figure S6), compound **7** is most often located at the tip of the regulatory head of unphosphorylated G159D, inserted between the extreme N terminus of cTroponin C and the switch peptide of cTroponin I, consistent with the CPPTraj data in Figure 4, whereas silybin B has been shown to be inserted between the N terminal region of cTnC and the N terminal peptide of cTroponin I.

This analysis is supported by the direct observation of trajectories. Observation confirms the stochastic nature of the ligand binding. A ligand can bind at several discrete locations with the site identified by cluster analysis predominating, although the ligand moves around in this site. In general, the ligand goes to one location and stays there for prolonged periods; however, the ligand can move during a run, either by “creeping” across the troponin or more often by dissociation and rebinding. Figure 5 shows Snapshots from MD trajectories showing compound **7** binding at its most probable location to G159D troponin; full trajectories may be viewed in Figure S7. Apparent contacts with the tail of troponin (around the TnI helix 1-helix 2 turns and the linker before TnT helix1) are observed but are likely to be artifactual since these parts of troponin will be interacting with the rest of the thin filament *in vivo*.

**Figure 5:**
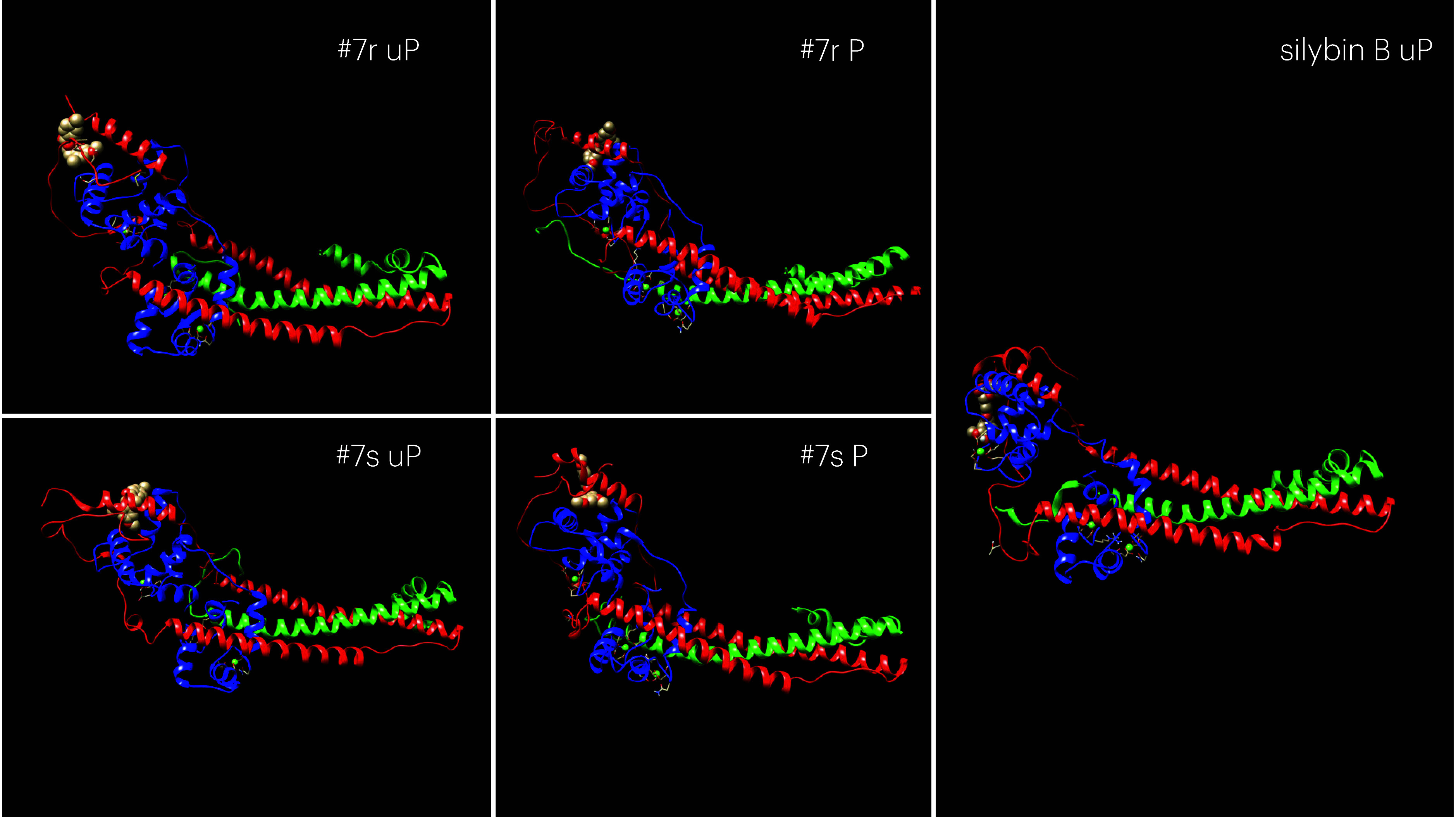
Snapshots from MD trajectories showing compound **7** binding at its most probable location to G159D troponin. The full trajectories are shown in Figure S7. The location is similar for both r and s isomers and is also the same in phosphorylated and unphosphorylated states. For comparison we show unphosphorylated silybin B, whose most probable binding position is intercalated between the N terminal peptide of TnI and helix B-C linker region of troponin. This site is not occupied in phosphorylated troponin. Blue, Tni: green, TnT: Red, TnI. Compound **7** is represented as spheres.

## Conclusions

Phosphorylation of cardiac troponin by protein kinase A plays a key role in the heart’s response to adrenergic stimulation. Phosphorylation of cardiac troponin I leads to a 2-fold decrease in Ca^2+^-sensitivity of the myofilaments linked to a 2-fold increase in the rate of Ca^2+^-release from troponin and hence quicker relaxation. Mutations that cause cardiomyopathy suppress this regulation (uncoupling)^5,6^ and thus contribute to the disease phenotype.^6^ We have previously found a number of compounds related to EGCG and silybin that can restore the wild-type response (recouplers) as potential treatments.^9^ We also found one unique compound that did the opposite. 5,7-dimethoxychroman-3-yl 4-methoxybenzoate (compound **7**) causes mutant thin filament Ca^2+^ sensitivity to *increase* when cardiac troponin I is phosphorylated. This “reverse-recoupling” effect is observed with three thin filament mutations so it is likely that compound **7** acts similarly on all uncoupled species as was shown for silybin B and EGCG.^9^ Interestingly, compound **7** has no effect on the modulation of troponin by phosphorylation in wild-type thin filaments, in common with most recoupling molecules.

The unique functional effects of compound **7** are reflected by a correspondingly unique effect on mutant troponin dynamics and ligand binding compared to recouplers and desensitizers calculated from molecular dynamics simulations. The effect of compound **7** on G159D phosphorylation-dependent MD metrics is neither restoration of wild-type values as in recouplers nor zero effect as in desensitizers (Figure 3, Table 3, and 4 Figure S5).^10^ Instead, we see a tendency for the values to be independent of phosphorylation. The direct observation of ligand binding location and the clustering analysis also show a unique location and minimal dependence on phosphorylation (Figure 5, Figure S6).

This study indicates some important principles for the study of small molecule interactions with troponin. First, the demonstration of a third nonphysiological effect of small molecules on troponin indicates a degree of plasticity of troponin function not previously appreciated. The ability of similar compounds such as compounds **7**, **9**, or **12** to have different functional effects on troponin also shows great specificity in the consequences of ligand binding that could be of use in designing better compounds for recoupling.

Second, this study has shown, as was indicated by previous studies, that effects of small molecules, demonstrated *in vitro*. correlate with effects on troponin dynamics and ligand binding calculated by molecular dynamics simulations and thus validates this molecular dynamics methodology for further study of small molecule effectors of troponin *in silico*.

## Materials and Methods

Reagents were obtained from Sigma-Aldrich, except for silybin B that was prepared by Prof. Vladimir Kren and Dr. David Biedermann.^14^ Compounds **7**, **9**, **12**, and **19** were synthesized by Anuj Khandelwahl.^11^

The movement of synthetic thin filaments over immobilized fast skeletal muscle HMM was measured by *in vitro* motility assay as previously described.^15^ The fraction of filaments motility and the filament velocity were measured. The fraction motile parameters are plotted here. We measured Ca^2+^ activation curves for thin filaments containing native human cardiac troponin, skeletal muscle actin, and wild-type or E180G mutant tropomyosin (tpm1.1) in the unphosphorylated and phosphorylated states obtained by phosphatase and kinase treatments.^12^ A single Ca^2+^-concentration assay was used as a rapid screen for the coupling and recoupling activity and dose–response curves. [Ca^2+^] was 0.073 μM as previously described.^9^

### Molecular Dynamics Studies

MD simulations of troponin were performed as described by Yang et al.^16,10^ The cTn model used for simulations in this project was based on that constructed and extensively studied in the group.^17,16,18^ The phosphorylated systems and G159D mutation were created from this model by modifying Ser22, Ser23 of the cTnI protein, and Gly159 of cTnC by manually editing the residues. The computational model was prepared in two different phosphorylation states: uP (no phosphorylation) and SEP (cTnI S22/S23 phosphorylated with net charge −2 each). All simulations started with the same structure. Ligands were added to the solvent 10 Å away from the troponin surface.

All simulations were performed using the AMBER18 software suite^19^ and the CUDA accelerated PMEMD^20^ code in the SPFP precision mode.^21^ All parameter and topology files were prepared using LEaP, which is part of AmberTools19 with AMBER ff14SB force-field library for amino acids and phosaa10 parameter library for phosphoserines (SEP).^22,23^

The simulation systems were set up by placing the protein complex in a cubic periodic boundary box with an edge length of 140 Å. The systems were first neutralized by adding Na^+^ ions as counterions then solvated by adding water molecules (TIP3P water model^24^). To simulate physiological conditions, water molecules were replaced by ions at random to achieve a concentration of approximately 0.15 M NaCl. Each system underwent a 5-stage minimization, heating, and equilibration process before production runs:

1. An initial minimization of the solvent for a maximum of 10000 cycles and the protein atoms were restrained with a force constant of 10000 kcal mol^–1^ Å^–2^. The minimization algorithm was switched from steepest descent to conjugate gradient after 500 steps.
2. A second minimization of all atoms in the system for a maximum of 2500 steps and switching from steepest descent to conjugate gradient after 1000 steps.
3. A first-stage heating from 0 to 100 K under an NVE ensemble with weak restraints on the protein complex (10 kcal mol^–1^ Å^–2^), except the N-terminal of TnI (1–32). The temperature was increased linearly over 50 ps.
4. A second-stage heating from 100 to 320 K over 60 ps, followed by a cooling from 320 to 300 K over 40 ps under an NPT ensemble.
5. An equilibration step of the system for 10 ns with an NPT procedure.

The minimization steps were done under constant volume with a nonbonded interactions cutoff of 12 Å. The NPT ensemble was controlled through Langevin dynamics with a collision frequency of 1 ps^–1^. A Monte Carlo barostat was used to maintain a 1 bar pressure with a relaxation time of 1 ps^–1^. For the heating, equilibration, and production runs, a nonbonded interactions cutoff of 8 Å was employed and the SHAKE algorithm^25^ was used to constrain hydrogen bonds to allow for a larger time step. Hydrogen mass repartition was employed for the equilibration and production steps to allow a 4 fs time step.^26^ Five repeats were done for each system’s production simulation, and each production run lasts 1500 ns.

Parameterization of the ligands in this study was based on a general AMBER force field (GAFF), which is suitable for small organic molecules.^27^ Ligand partial charges were derived from the ground state structures which were found by following conformational search, geometry optimization, and thermal correction procedures, as detailed below: An ensemble of conformers was generated for each ligand using the ETKDG algorithm as implemented by RDKit.^28^

Each conformer was optimized to a local minimum. Each optimized structure was subjected to thermal correction with frequency to calculate the Gibbs free energy.^29–31^ The structure with the lowest ground-state energy was picked as the ground-state structure.

From the ground state structures, restrained electrostatic potential (RESP) calculations were used to obtain the charge distribution on the molecule.^32^ Gaussian16 ^33^ was used for both geometry optimization and RESP calculation. Geometry optimization was done with B3LYP functional with cc-pVDZ basis set and the polarizable continuum model (PCM) in water as an implicit solvent.^34–37^ RESP calculation was done with the Hartree–Fock method and 6-31G* basis set in a vacuum.^38^ AmberTools was then used to generate the charge parameters for each atom.

Binding energy estimation by MMBPSA, interhelical angle distribution, and hinge angle analysis was performed as previously described.^16^ CPPTraj was used to determine the contact/interactions. Pytraj,^39^ a Python package binding to cppraj program,^40^ was used for the distance measurements in this work. The ligand was deemed to be in contact with a residue when the minimal distance between any atom of the ligand and a residue was less than 2.5 Å.

Cluster analysis was performed as described by Yang.^18^ To identify clusters of ligand positions, the simulated conformers were first superposed to their respective representative structure. This ensures that the ligands were in the relative position to the same protein conformer. The pairwise distance between the center of mass of each ligand position was then calculated. Density-based spatial clustering of applications with noise (DBSCAN) procedure as part of scikit-learn was used to find the clusters.^41^ Eps, the maximum distance between two ligands’ center of mass to be considered neighbors, was set to 10 Å, and it required a minimum 1% of the total number of frames to form a cluster.

## Supplementary Material



## Notes

S1: **19**, **12**, and **7** dose–response curves at 0.74 μM Ca^2+^, G180D TM WT Tn S1a Motile velocity plots corresponding to % motile in Figures 2 and S1. S2: Silybin and **7** dose–response curves at 0.74 μM Ca^2+^, TPM E54K, or ACTC E99K-containing thin filaments. S3A: Structures and chemical names of the compounds studied in this paper. S3B: Preferred solution conformation of **7s** is superimposed on EGCG and SilybinB. S4: Distribution of interdomain hinge angle, TnC helix A/B angle, and interdomain distance determined by molecular dynamics simulations. S5: Quantification of the effect of phosphorylation and small molecules on interdomain distance and on the interdomain ionic bond between TnC G/D159 and R83. S6: Binding site clusters for systems with compounds **7r** and **7s**. S7: Molecular dynamics trajectories for compound **7** interacting with G159D troponin. (PDF)

## Abbreviations

IVMA: *In vitro* motility assay
G159D: Troponin with G159D mutation in troponin C
EGCG: Epigallocatechin-3-gallate
MD: Molecular dynamics
fwhm: Full width at half-maximum
compound **7**: 5,7-Dimethoxychroman-3-yl 4-methoxybenzoate
9: 5-Hydroxychroman-3-yl-4-methoxybenzoate
12: 5,7-Dihydroxychroman-3-yl-3-hydroxybenzoate
19: 5,7-Dimethoxychroman-3-yl 3,5-dimethoxybenzoate
cTnI: Cardiac troponin I
cTnC: Cardiac troponin C
