# Supplementary material for: 5,7-Dimethoxychroman-3-yl 4‑methoxybenzoate Has a Unique Effect upon the Modulation of Mutant Cardiac Muscle Thin Filament Function and Dynamics due to Phosphorylation of Troponin I
Source: ACS Pharmacol Transl Sci. 2025 Apr 15;8(5):1251–9. doi: 10.1021/acsptsci.5c00156 (PMC12070315; doi:10.1021/acsptsci.5c00156)
Supplement: Supplementary file 1 [file pt5c00156_si_001.pdf]

Supporting Information

5,7-Dimethoxychroman-3-yl 4-Methoxybenzoate has a unique effect upon the modulation of mutant cardiac muscle thin filament function and dynamics by phosphorylation of troponin I

Zeyu Yang‡, Alice M Sheehan,‡ Mary Papadaki, Andrew E Messer, Brian SJ Blagg, Ian R Gould, Steven B Marston*

10 Pages , 7 Figures and Tables

Figure S1

19, 12 and 7 dose-response curves at 0.74μM Ca2+, G180D TM WT Tn

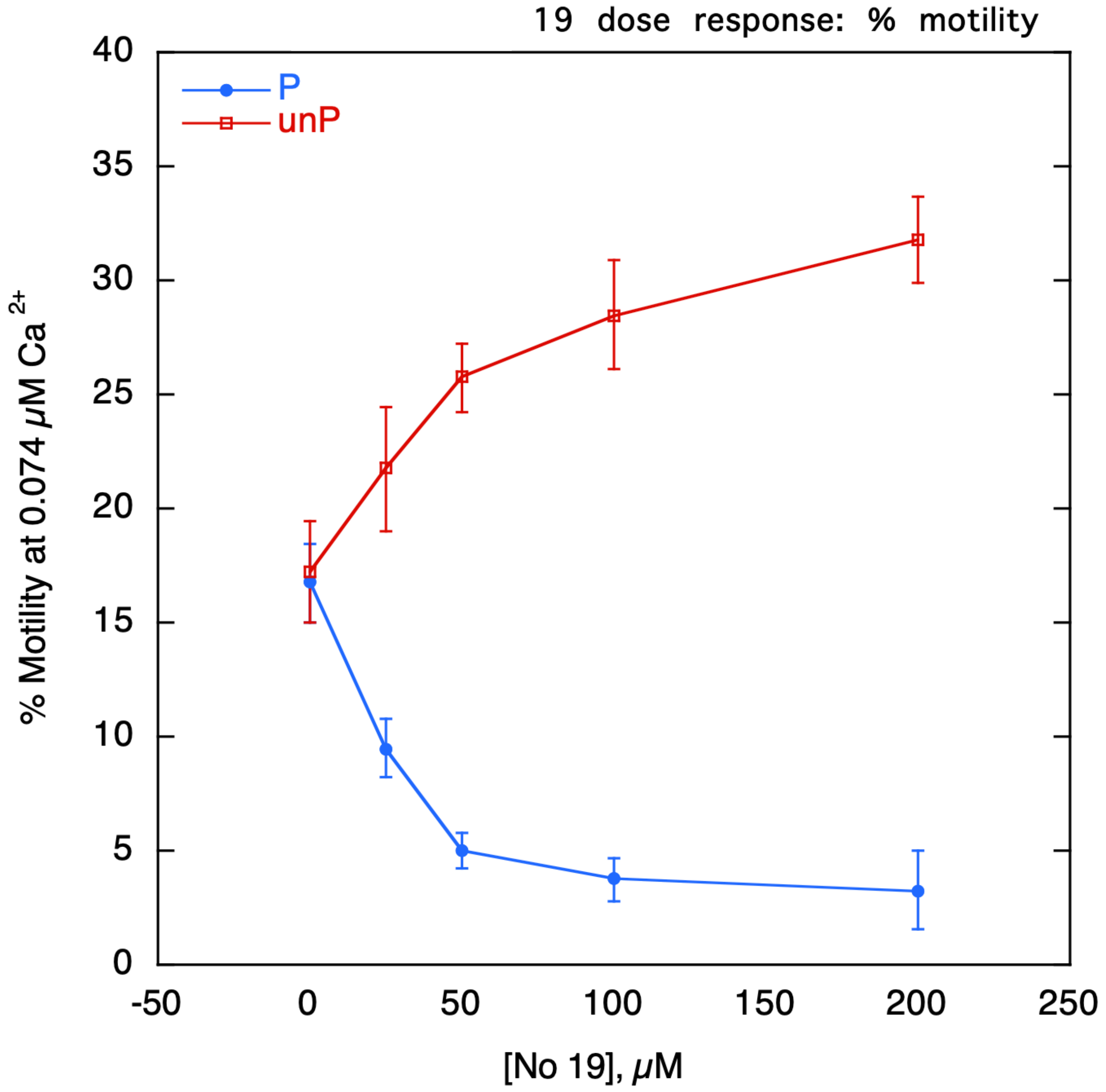

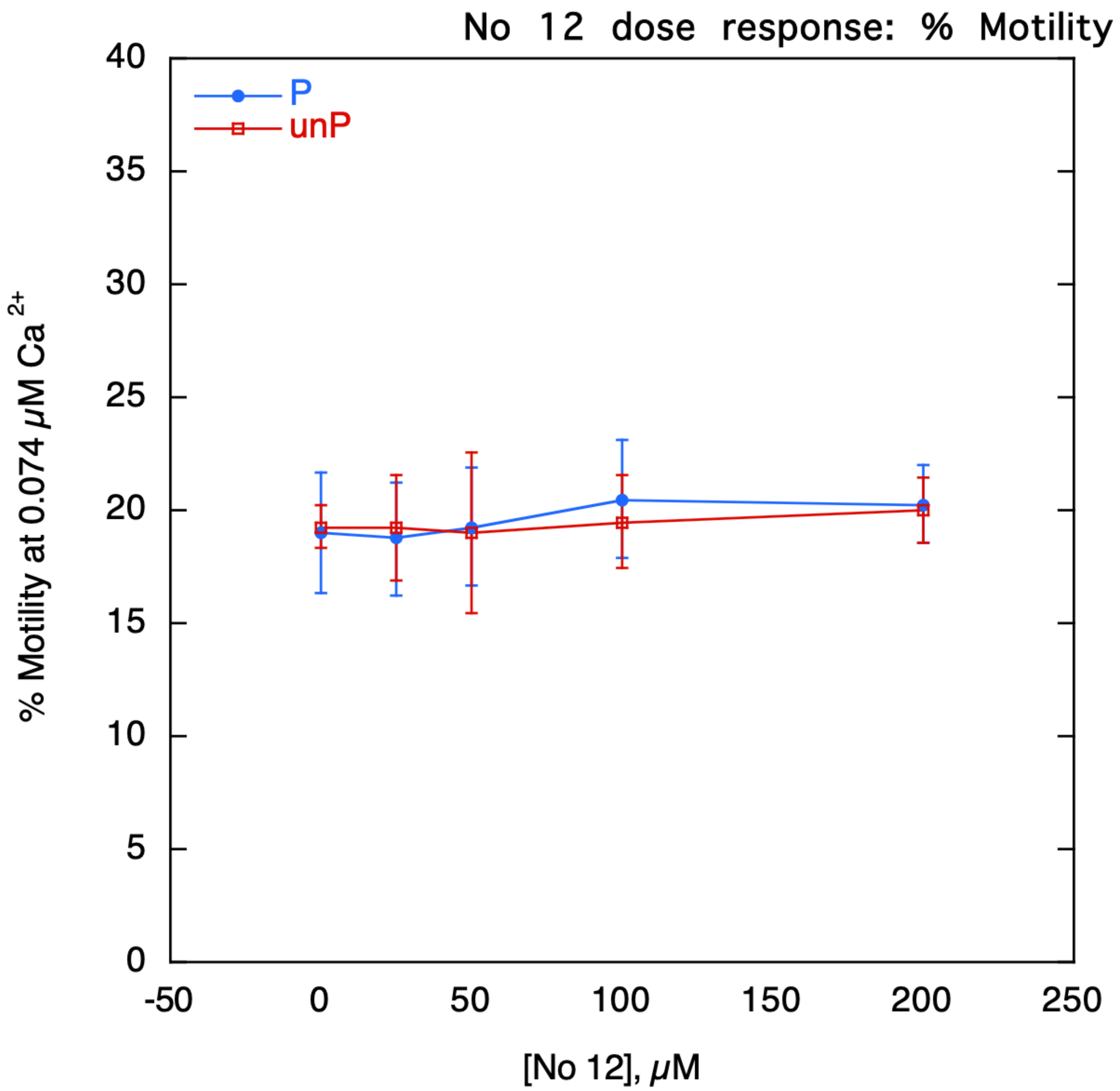

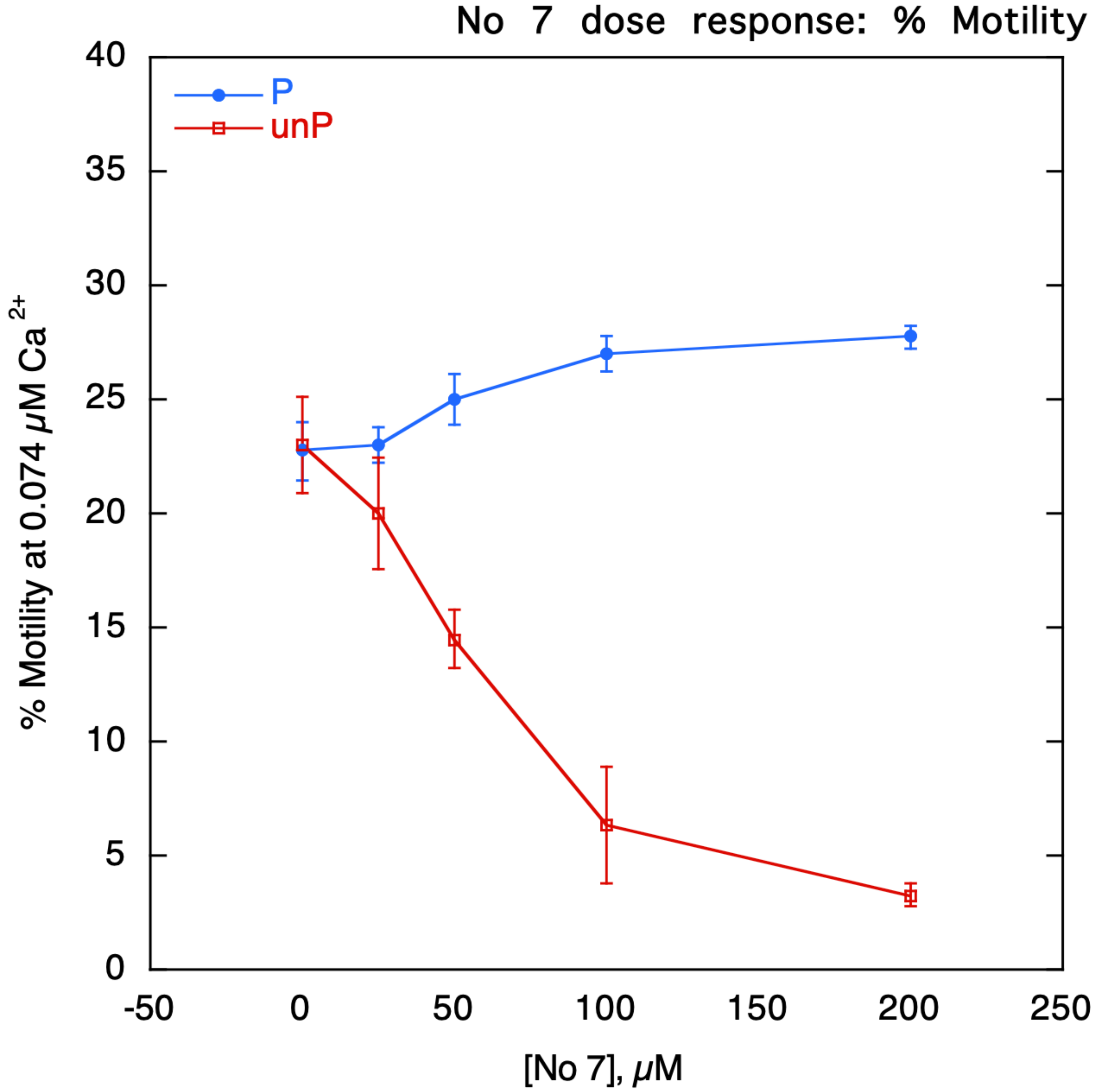

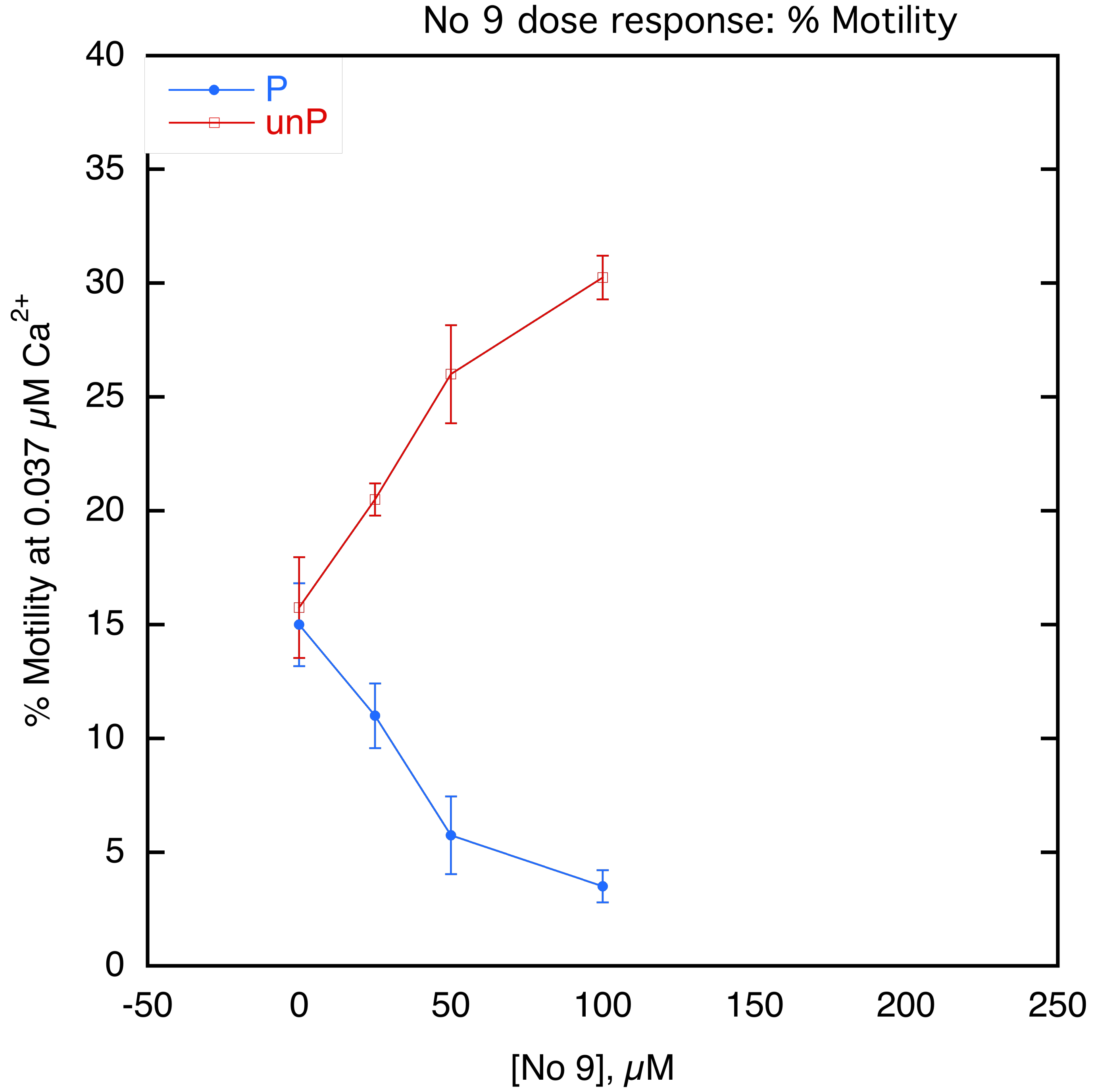

Single [Ca2+] motility assay: 19, 12, 9 and 7 dose-response curves at 0.74μM Ca2+, G180D tropomyosin + WT actin and troponin.

19 and **9** are normal recouplers, **12** has no activity as a recoupler and **7** has a reverse effect
Most small molecules act like **19,** e.g. Silybin B and Resveratrol, or **12** (inactive) so **7** has a unique property and induces an unphysiological state of troponin

Figure S1a

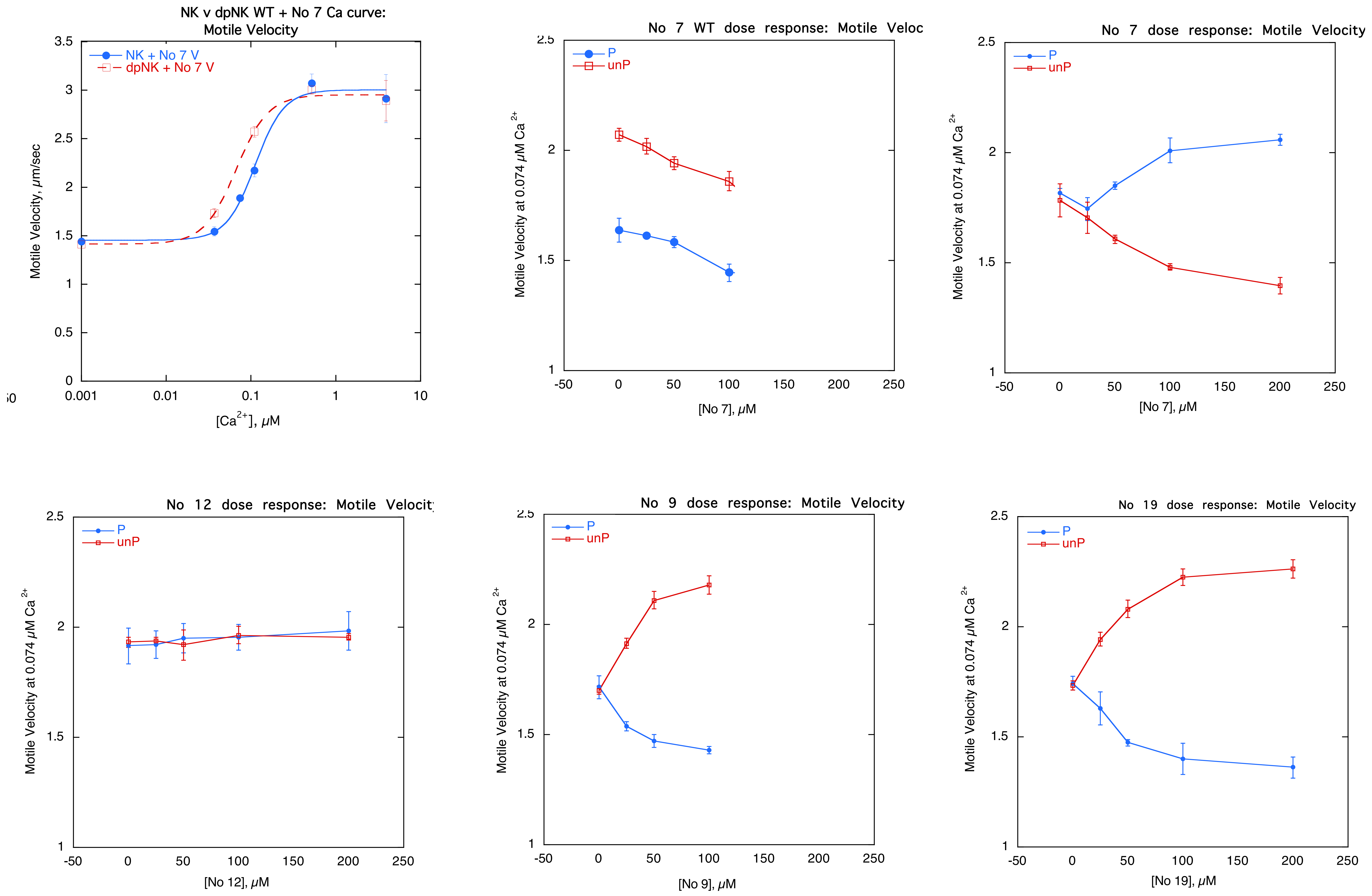

Motile velocity plots corresponding to % motile in manuscript and Supplement 1

Figure S2

Silybin and 7 dose-response curves at 0.74μM Ca2+, TPM E54K or ACTC E99K -containing thin filaments

TPM1 E54K
thin filaments

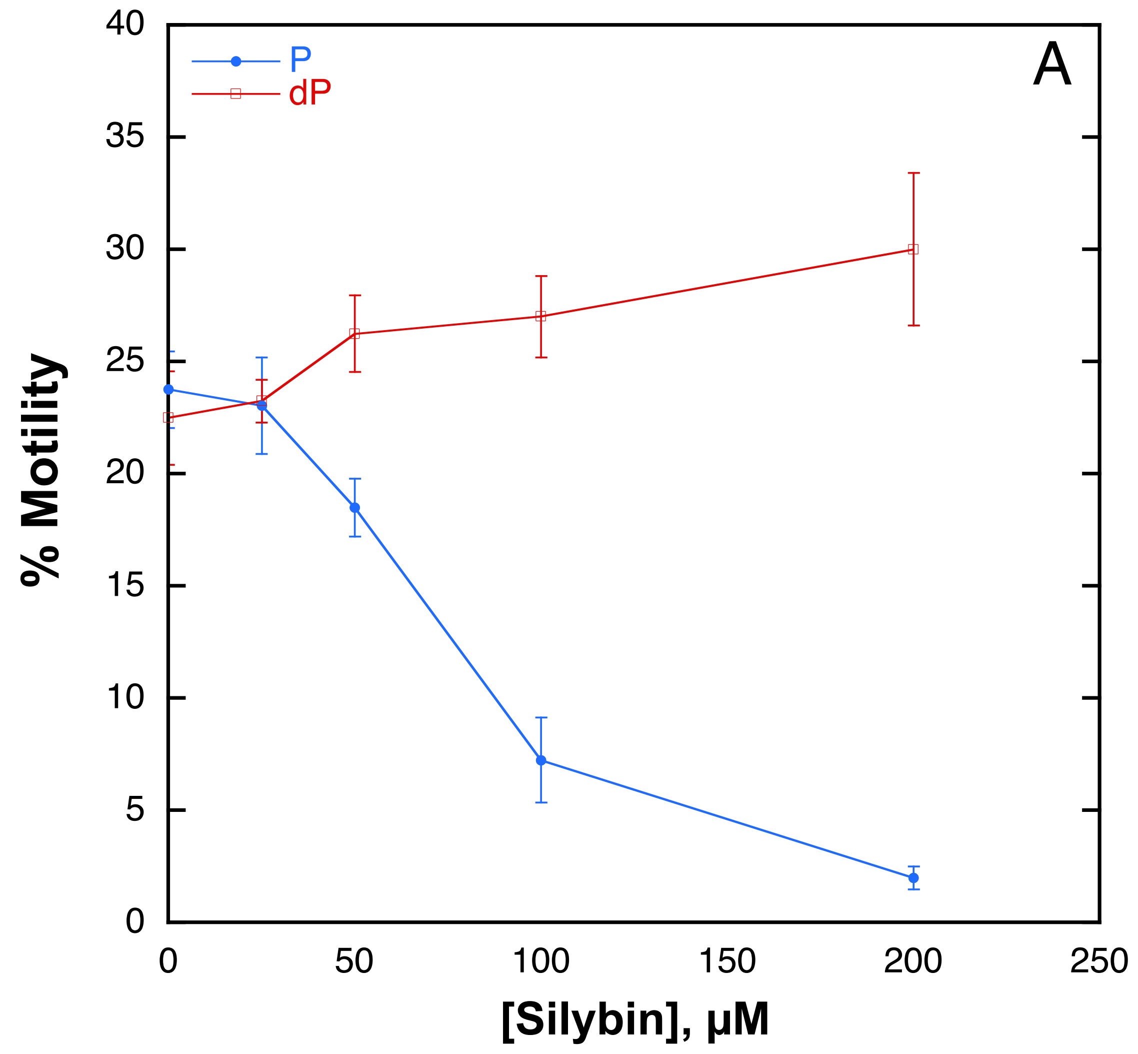

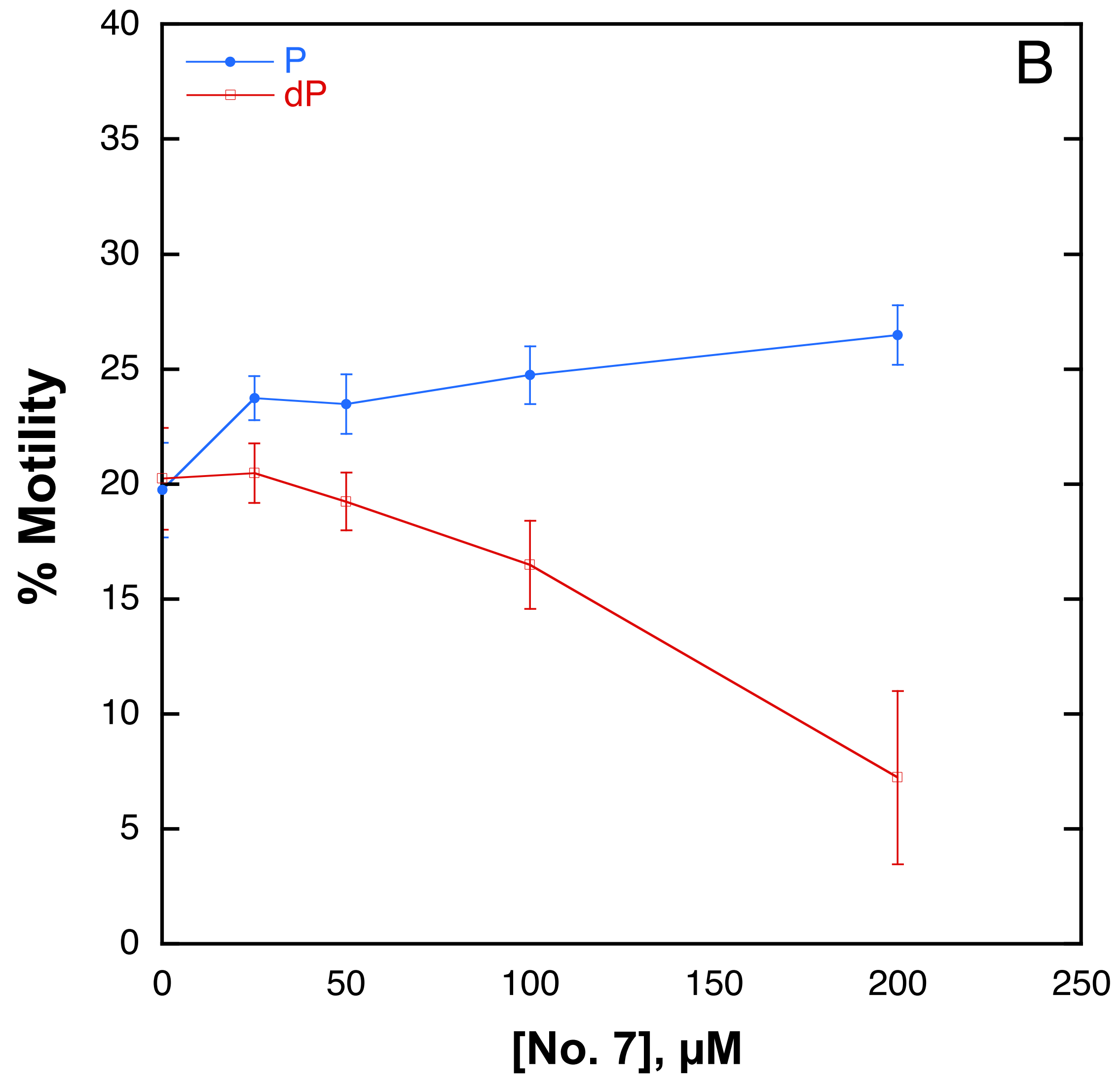

ACTC E99K
thin filaments

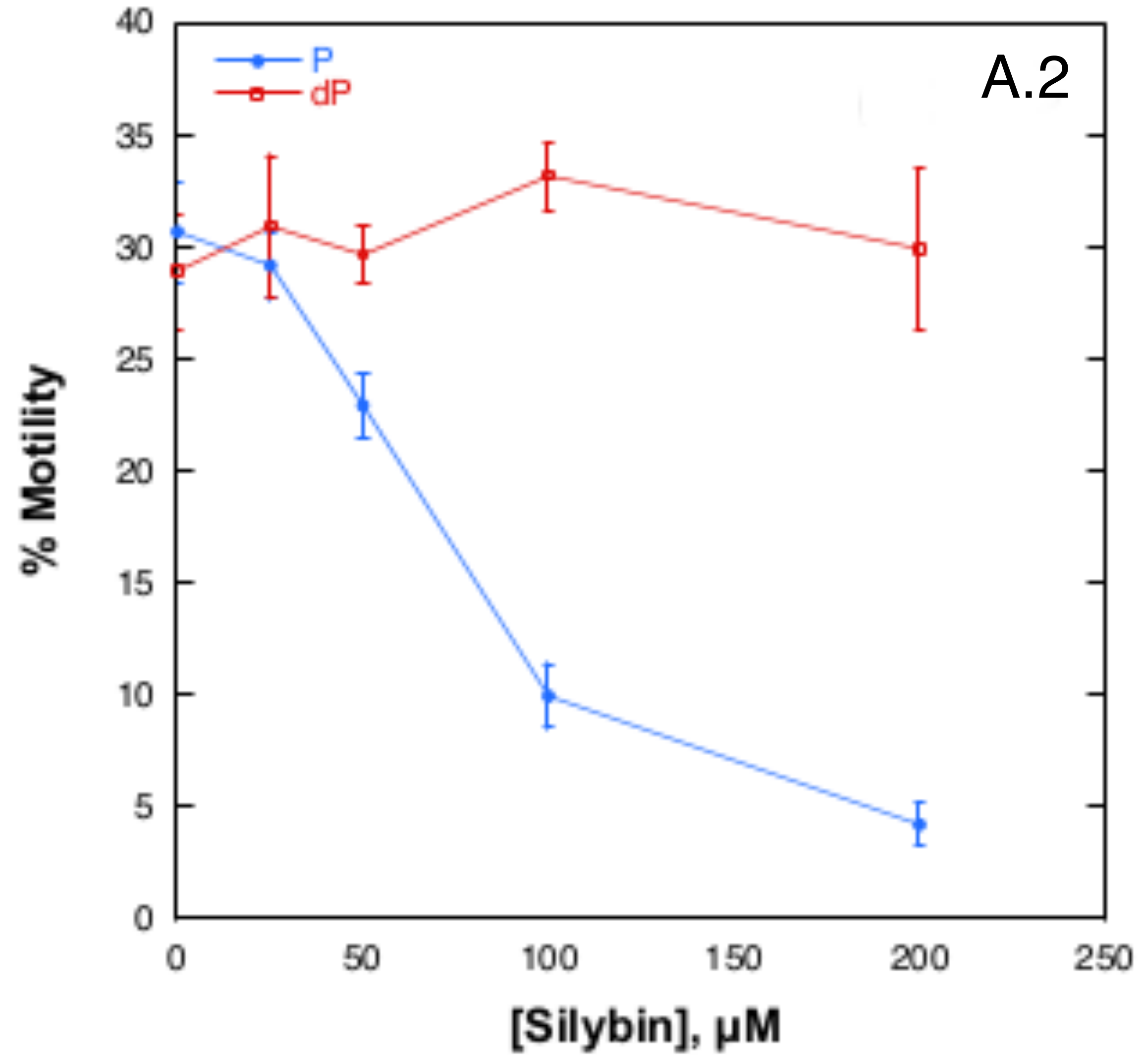

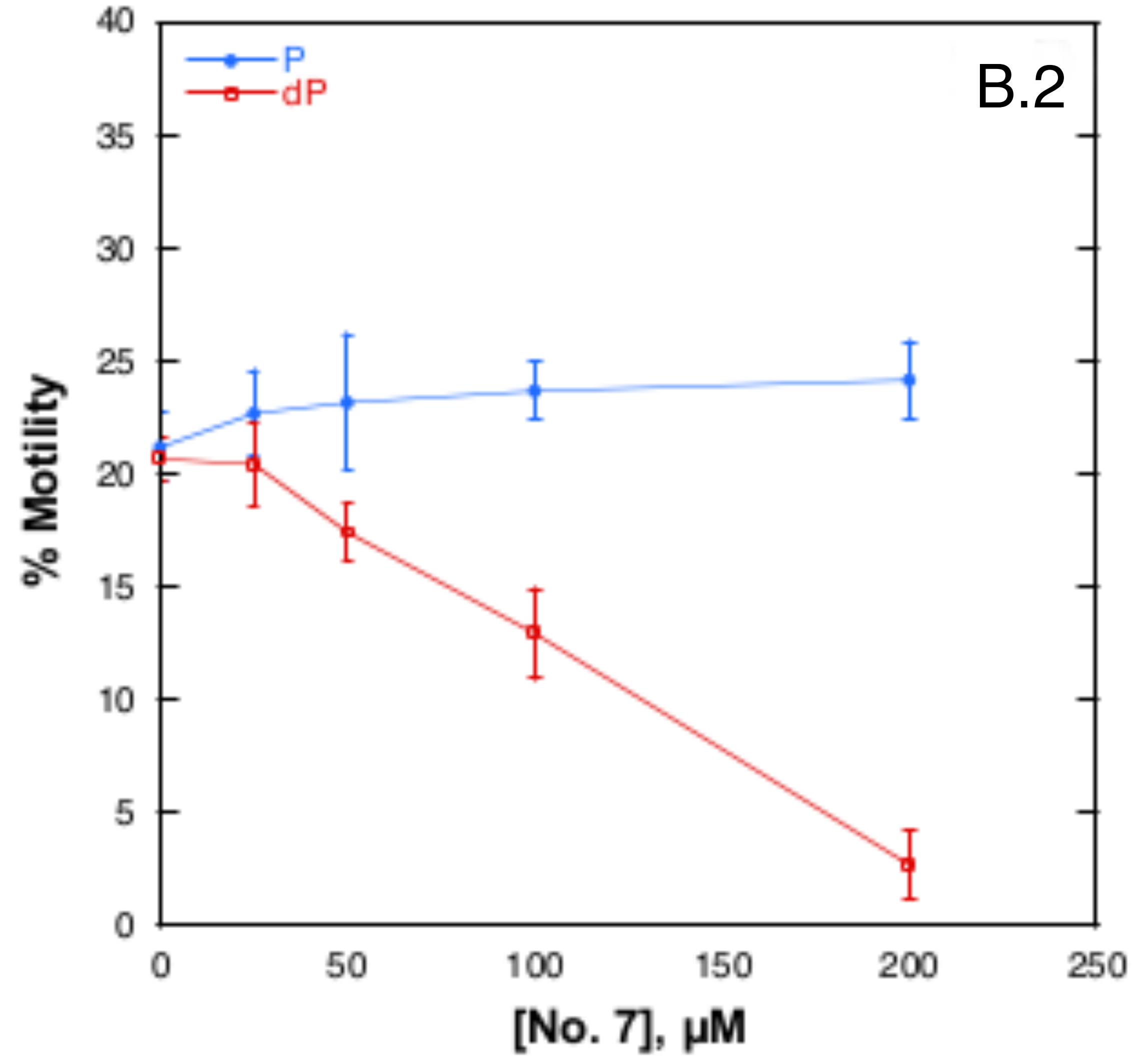

Figure S3A

S3A Structures and chemical names [illegible] the compounds studied in this paper

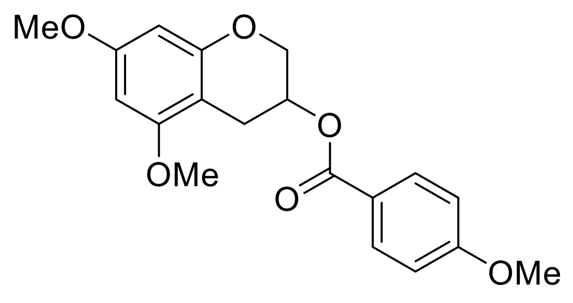

7

5,7-Dimethoxychroman-3-yl 4-Methoxybenzoate

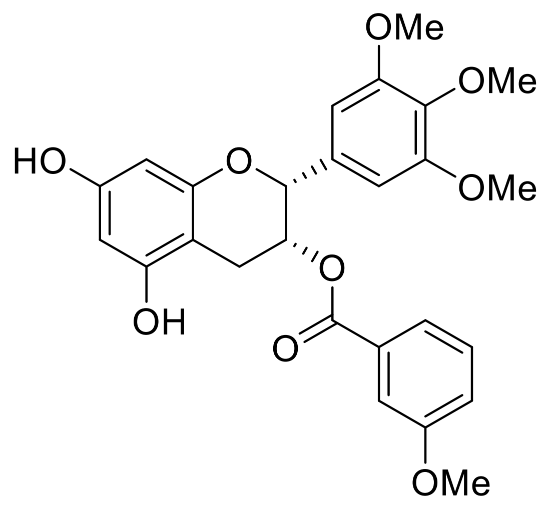

Epigallocatechin-3-gallate (EGCG)

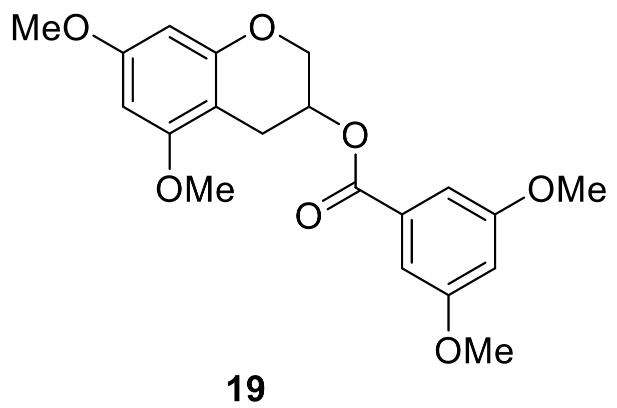

19

5,7-Dimethoxychroman-3-yl
3,5-Dimethoxybenzoate

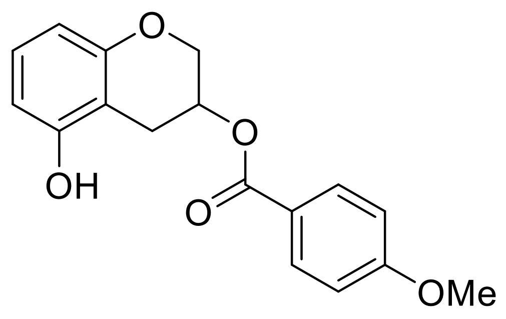

9

5-Hydroxychroman-3-yl-
4-Methoxybenzoate

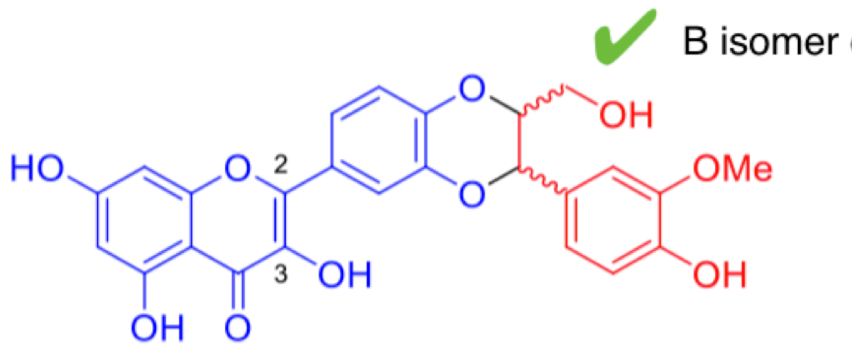

Silybin B

Recouplers

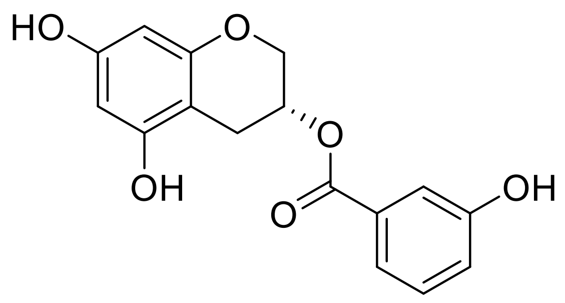

12

5,7-Dihydroxychroman-3-yl-3-Hydroxybenzoate

Figure S3B

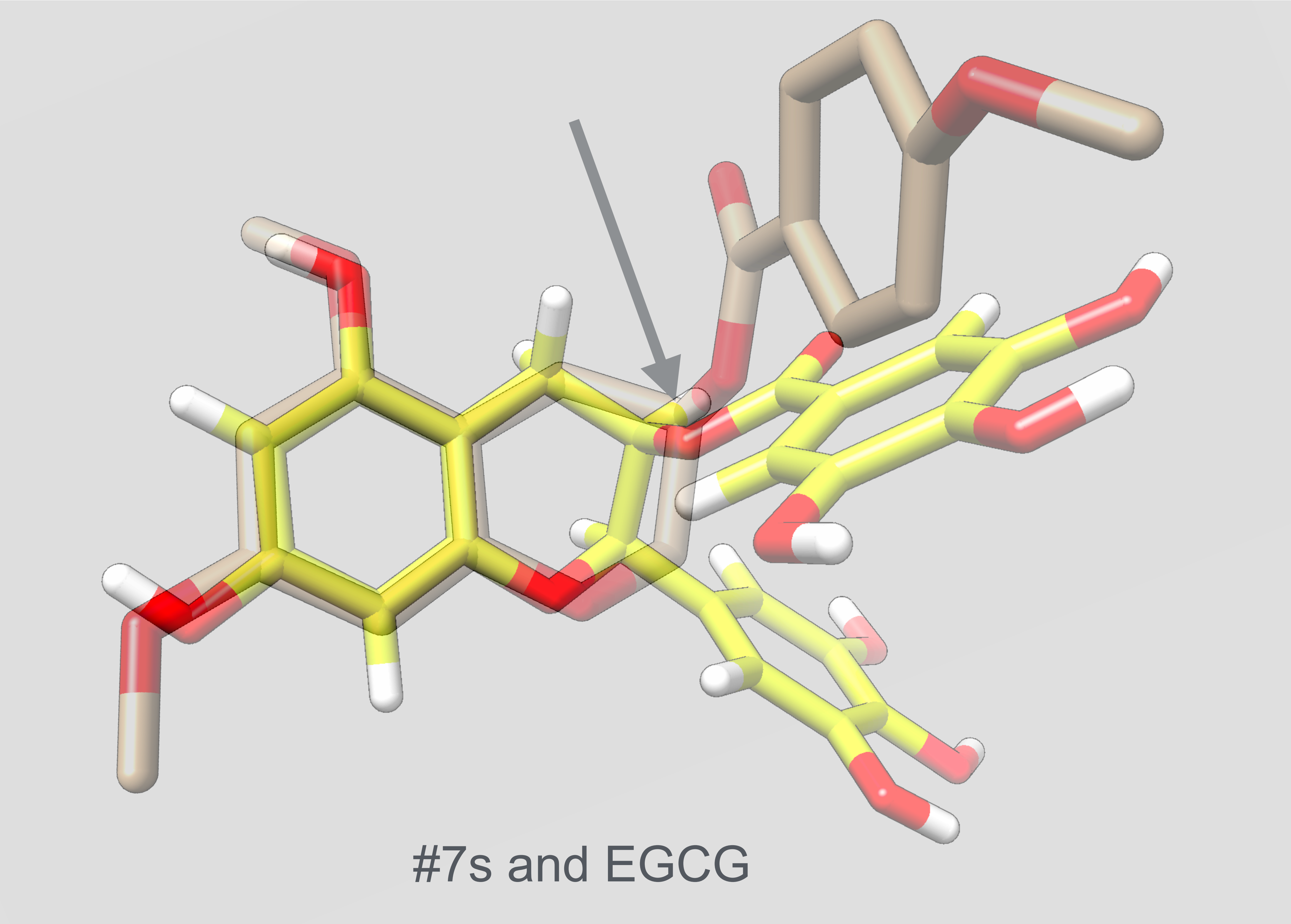

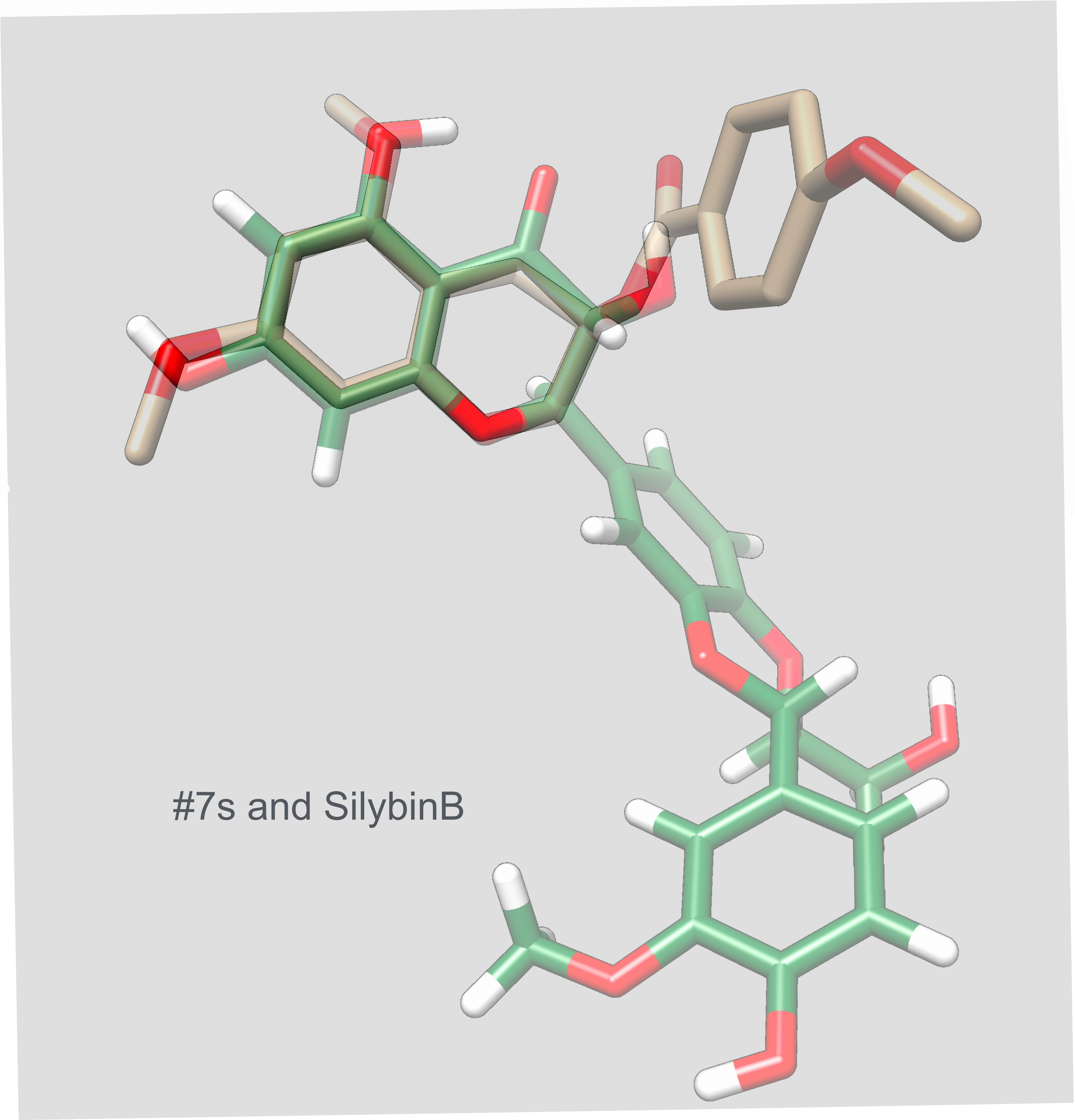

The preferred solution conformation of #7s is superimposed on EGCG and SilybinB

Figure S4

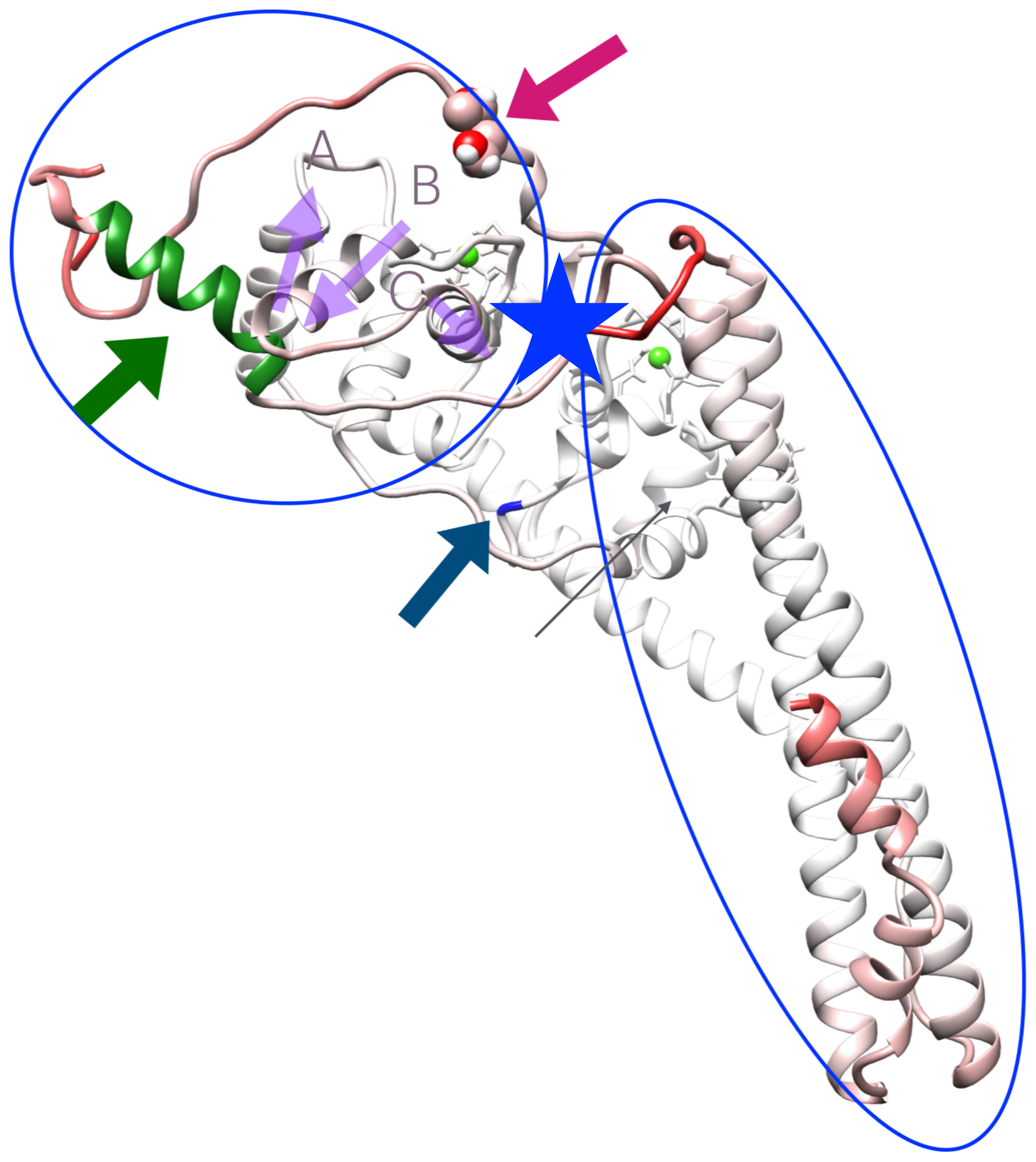

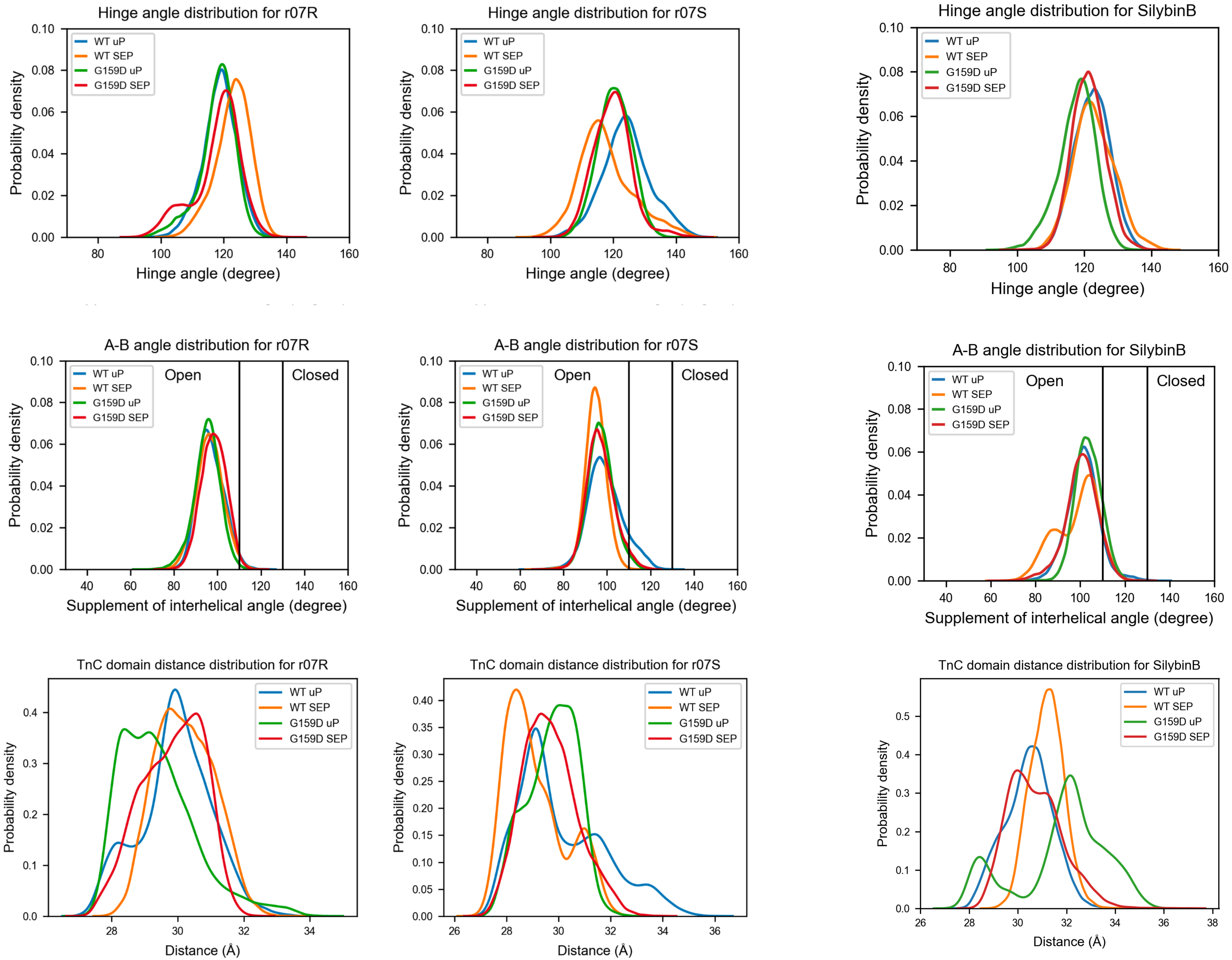

The distribution of interdomain hinge angle, TnC helix A/B angle and interdomain distance determined by molecular dynamics simulations.

Left :Model defining the location of the hinge between N-terminal and C-terminal domains of troponin C (blue star) and the orientation of helices A and B in the N-terminus of troponin C (purple arrows). TnI Serines 22 and 23 are shown as spheres indicated by red arrow. TnC G159 is shown (blue arrow) and TnI switch peptide is shown in green (green arrow). Peptide mobility is shown by red shading.

The effects of the G159D mutation and phosphorylation on the distribution of the interdomain angle, the helix A/B angle and interdomain distance in the presence of #7R, #7S and silybinB is plotted. Unphosphorylated, uP, phosphorylated, SEP.

Figure S5

Quantification of the effect of phosphorylation and small molecules on interdomain distance and on the interdomain ionic bond between TnC G/D159 and R83.

			Interdomain distance metrics								TnC D159-R83 ionic bonding	
			Mean distance, Å				FWHM, Å				%	
troponin	ligand		uP mean (std)	Δ	P		uP	Δ	P		uP	P
WT	apo		30.18 (1.34)	-0.28	29.9 (0.9)		2.99	-0.61	2.38		0	0
G159D	apo		29.22 (0.98)	0.35	29.59 (1.18)		1.57	-0.06	1.51		80	76
G159D	EGCG		31.17 (1.48)	0.81	31.98 (1.59)	?	2.38	-0.57	1.81	✅	22	53
G159D	SilybinB		31.83 (1.83)	-1.11	30.72 (1.13)	✅	1.9	0.74	2.64	?	17	22
G159D	#7r		29.4	0.4	29.8		2.6	0	2.6		85	72
	#7s		29.8	-0.1	29.7		2.6	-0.2	2.4		48	70

Figure S6

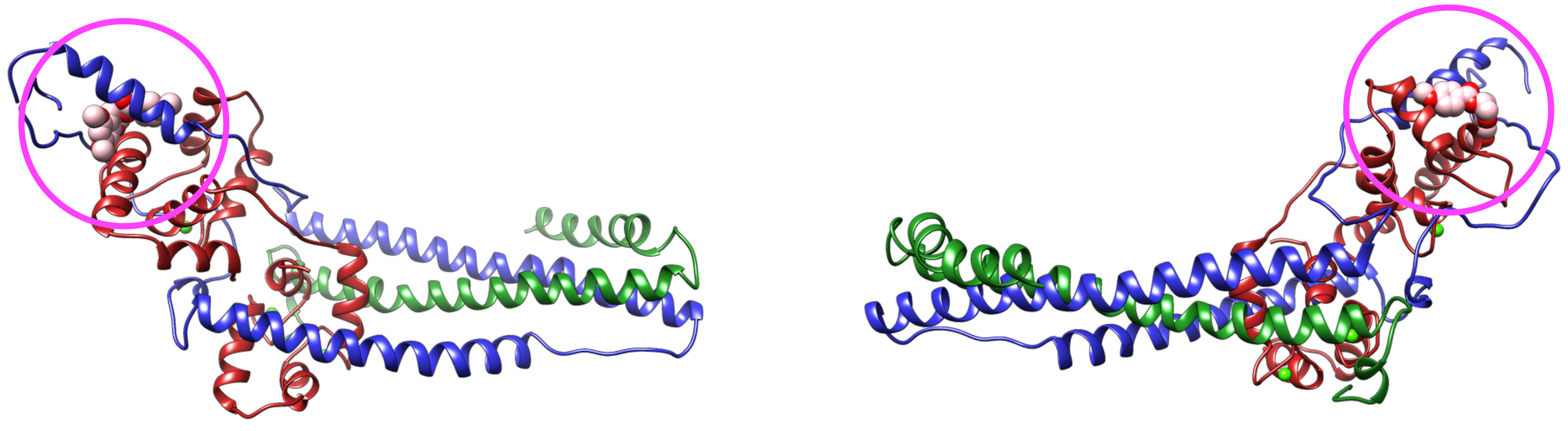

G159D uP

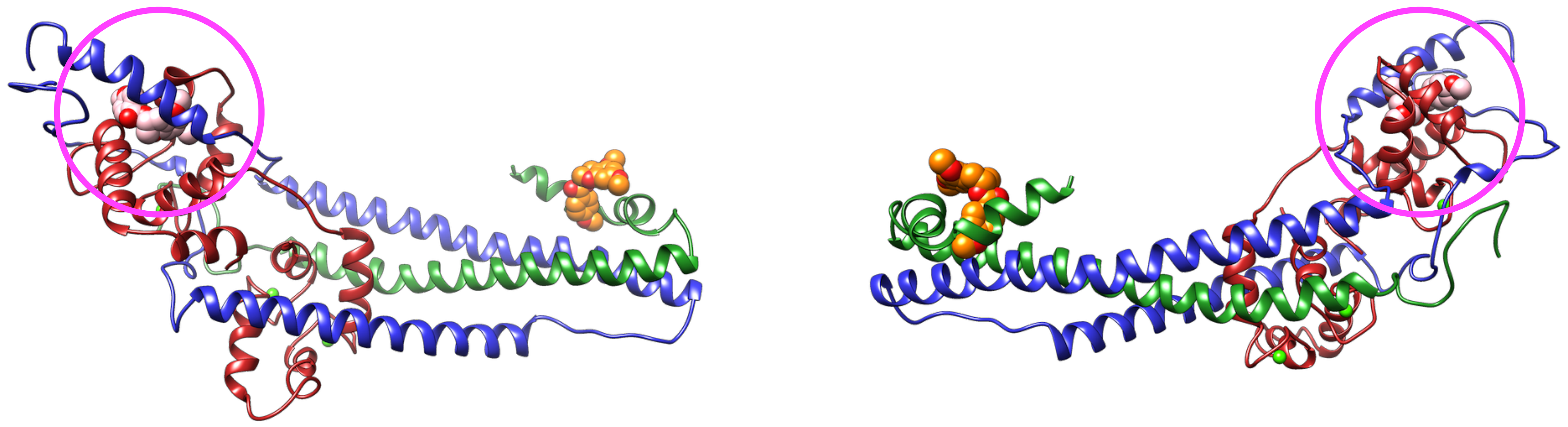

G159D SEP

Binding site clusters for systems with r07R.

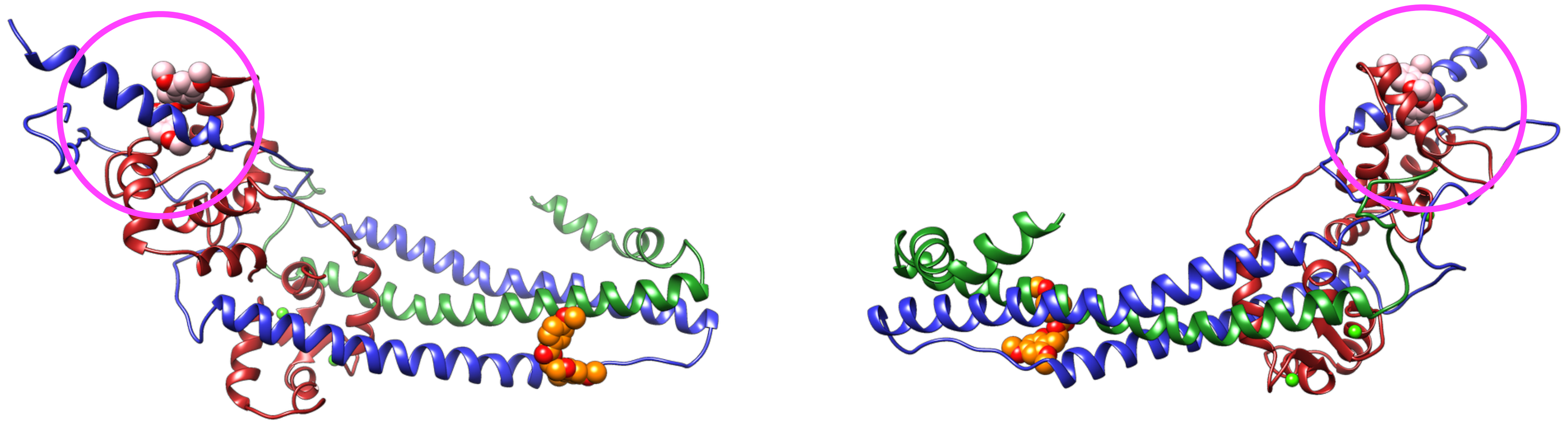

G159D uP

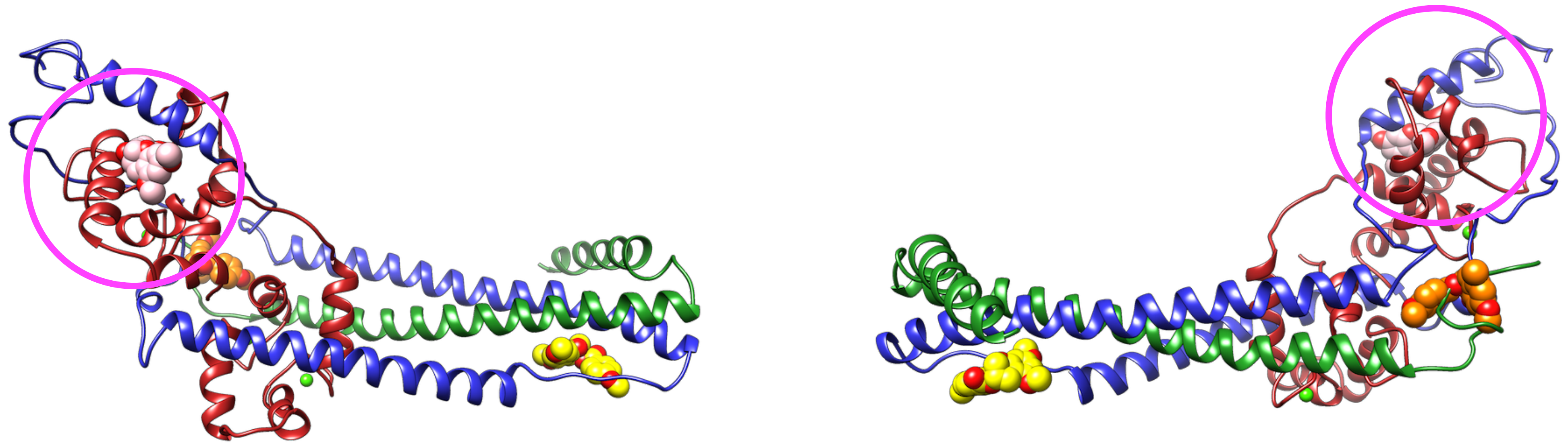

G159D SEP

Binding site clusters for systems with r07S.

Figure S7

Molecular dynamics trajectories for compound 7 interacting with G159D troponin.

Five runs of 1.5µs were run for each condition (7500 frames). The runs are shown as .mov files on this link.

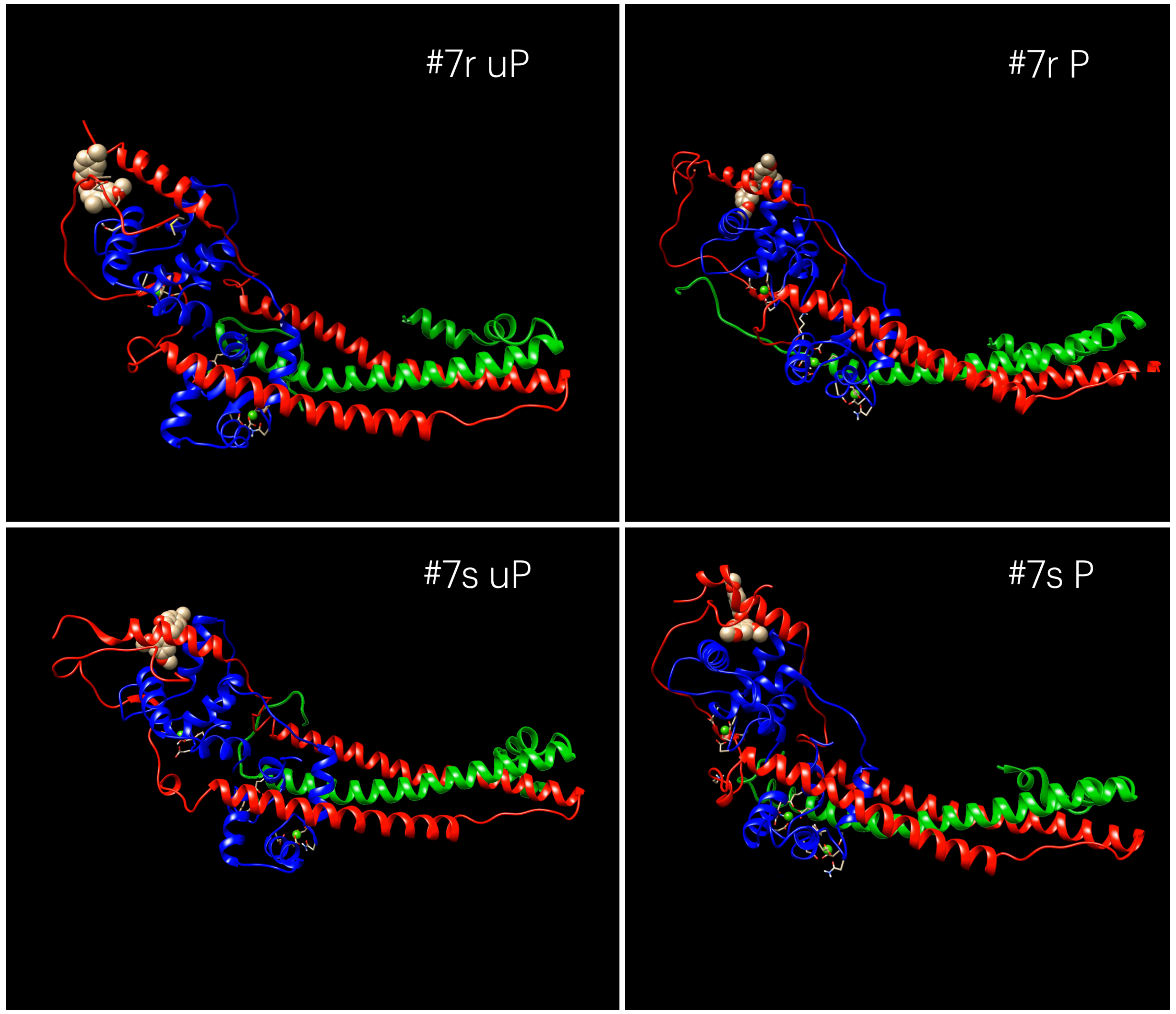

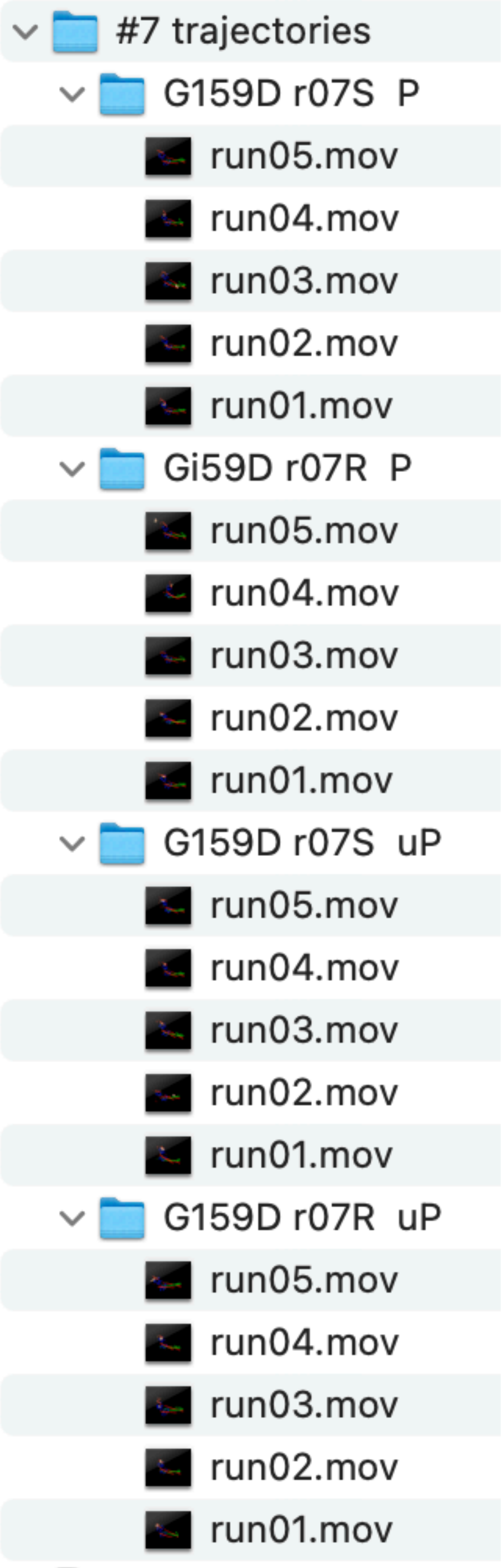

https://www.dropbox.com/scl/fo/vhqc8k6tyvwxj5mifa4dk/APCNVbXzil4iRm06S_sscGo?rlkey=dzp5qxc2bs6s0hxneribdcygp&dl=0
